# Healthcare providers' advocacy approaches and ethical challenges in delivering healthcare to undocumented migrants: a scoping review

**DOI:** 10.1007/s11019-024-10225-8

**Published:** 2024-10-07

**Authors:** Fayez Abdulrazeq, Julian März, Nikola Biller-Andorno, Chris Gastmans

**Affiliations:** 1 Institute of Biomedical Ethics and History of Medicine, Faculty of Medicine, University of Zurich, Winterthurerstrasse 30, 8006 Zurich, Switzerland; 2 Centre for Biomedical Ethics and Law, Faculty of Medicine, KU Leuven, Kapucijnenvoer 7, 3000 Louvain, Belgium

**Keywords:** Healthcare providers, Undocumented migrants, Health advocacy, Ethical challenges

## Abstract

Delivering healthcare to undocumented migrants presents a complex challenge for healthcare providers. Integrating advocacy efforts into their daily practices can be ambiguous in practical terms, stemming from the intricate task of addressing the health needs of this population while simultaneously advocating for their health rights within the constraints imposed on them. This study seeks to consolidate findings from literature regarding the advocacy approaches employed by healthcare providers and the correlated ethical challenges. We conducted a scoping review of qualitative literature by systematically searching four databases—PubMed/Medline, Embase, Cinahl, and Cochrane Library. For developing our search strategy, we employed the PICO (Population, Intervention, Comparison, Outcome) scheme. Our analysis followed the qualitative content analysis approach proposed by Graneheim and Lundman. 30 studies were included, revealing a cumulative total of 915 healthcare providers who were interviewed. A total of 30 themes emerged comprising 14 advocacy approaches and 16 ethical challenges. Healthcare providers made a deliberate choice to engage in advocacy, responding to injustices experienced by undocumented migrants. The spectrum of advocacy initiatives varied, encompassing voluntary participation in healthcare provision, empathetic understanding, and healthcare-focused strategies. We also identified numerous correlated ethical challenges, necessitating healthcare providers to strike a balance between their eagerness to assist and their professional competence, respect the autonomy of undocumented migrants, and establish trust with them. These findings not only offer practical guidance for healthcare providers to enhance accessibility to healthcare services for undocumented migrant patients but also foster awareness of the ethical challenges that may arise in their advocacy roles.

**Supplementary Information:**

The online version contains supplementary material available at 10.1007/s11019-024-10225-8.

## Introduction

The barriers to healthcare access for undocumented migrants are complex and have been explored in numerous studies, highlighting their significance and widespread impact (Hacker et al. 2015; Clifford et al. 2023; Winters et al. 2018). These barriers include policy-related challenges such as legal restrictions and insurance exclusions, health system obstacles like resource limitations, discrimination, and bureaucratic complexities, as well as individual impediments such as fear of deportation, language and cultural disparities, stigma, and limited understanding of the healthcare system in host countries (Hacker et al. 2015). Consequently, these barriers lead to the underutilization of healthcare services by undocumented migrants or the receipt of substandard care, even when services are available (Winters et al. 2018), thereby negatively affecting their physical and mental health (Magalhaes et al. 2010; Woodward et al. 2014).

Addressing these barriers and meeting the healthcare needs of undocumented migrants also pose a multifaceted challenge for healthcare providers. This challenge extends beyond the confines of individual care and provider-patient relationship dynamics to include organizational and societal constraints (Armin 2019; Tiedje and Plevak 2014). Owing to the constricted availability of healthcare services for this vulnerable population and the complication of their socio-economic needs, healthcare providers often grapple with the complexities of their roles (Straßmayr et al. 2012). They are compelled to confront multiple hurdles entrenched within the organizational framework and policy environment in order to ensure that their undocumented migrant patients receive fair and unbiased care, while simultaneously ensuring the continuation of the healthcare trajectory. To accomplish this, healthcare providers need to take on advocacy roles (Biswas et al. 2011; Fabi and Taylor 2019). However, incorporating health advocacy activities into everyday practices can be ambiguous and sometimes challenging in practical terms (Oandasan 2005; Earnest et al. 2010). Additionally, healthcare providers may have insufficient absorption of the ethical dimensions that determine a "good" advocacy practice, leading to uncertainty when confronted with ethical challenges as they endeavor to fulfill their advocacy roles (Berlinger and Raghavan 2013).

Health advocacy encompasses various concepts, levels, goals, and functions, serving as a crucial element in the pursuit of health promotion objectives. Advocacy initiatives can take place at the level of both “cases” and “causes”, aiming to safeguard the vulnerable through representational advocacy and empower the disadvantaged through facilitational advocacy (Carlisle 2000). Incorporating health advocacy into daily practice can be accomplished through two primary activities. The first involves "agency", where the focus is on promoting the health of individual patients by working the system. The second activity involves "activism", where the focus is promoting the health of community by changing the system (Dobson et al. 2012).^1^ Taking a more comprehensive view from an evolutionary model, health advocacy is seen as a deliberate action undertaken in support of both individuals and the community, particularly for those experiencing health inequalities (Farias et al. 2023).

Existing literature on advocacy for undocumented migrants has primarily focused on theoretical and conceptual frameworks (Dwyer 2004; Fabi 2019; Pallok and Ansell 2022; Acosta and Aguilar-Gaxiola 2014), while only a limited number of studies have emphasized the practical strategies employed by healthcare providers in their practices (Garcini et al. 2022; Morales et al. 2023). A gap in the literature exists regarding a comprehensive understanding of the advocacy approaches employed by healthcare providers serving undocumented migrants. This gap stems from a lack of systematic and scoping reviews consolidating evidence on this specific topic.

Moreover, while some papers have touched upon ethical challenges and their intersection with advocacy efforts for undocumented migrants (Berlinger and Raghavan 2013; Berlinger 2019; Kuczewski 2019), a systematic examination of these challenges and their implications for healthcare practice has been lacking. Therefore, to fill this gap in the literature and to assist healthcare providers in embracing their advocacy roles, we conducted a scoping review to investigate qualitative evidence regarding the advocacy strategies consistently employed by healthcare providers actively engaged in delivering healthcare services to undocumented migrants.

We aimed to understand how the integration of these advocacy practices empowered healthcare providers to deliver care to their patients, even in the face of organizational and systemic obstacles.^2^ Additionally, we sought to identify the correlated ethical challenges that emerged within this context. To achieve this goal, the following research question was formulated: What strategies (approaches) do healthcare providers employ to advocate for the health entitlements of undocumented migrants, and what ethical challenges arise in balancing individual patient needs with broader advocacy goals?

## Methods

### Design

In pursuit of our research objectives, we conducted a scoping review to identify and review relevant empirical qualitative research addressing the advocacy approaches of healthcare providers for undocumented migrants, along with ethical challenges within this domain. Considering the exploratory nature of our research question and its broad scope, we determined that employing a scoping review was the most suitable approach. The use of a scoping review allowed us to encompass a broad range of qualitative study designs and methodologies, facilitating the identification of knowledge gaps, particularly when the available literature is limited. To document this scoping review, we adhered to the guidelines outlined in the Preferred Reporting Items for Systematic Reviews and Meta-Analyses (PRISMA), specifically following the extension for scoping reviews (PRISMA-ScR) (Tricco et al. 2018).

### Inclusion and exclusion criteria

We conducted a thorough examination of a comprehensive range of qualitative empirical studies that explore the practical implementation of advocacy approaches or strategies by healthcare providers when delivering services to undocumented migrants. Our inclusion criteria were broad, encompassing studies where patient advocacy was not necessarily the primary research focus but was interwoven into the broader healthcare process. Before discussing our inclusion and exclusion criteria, we want to clarify key terms or concepts. For more information, please refer to Box 1.

**Box 1:** Key terms and concepts

**Healthcare provider:** Someone directly engaged in delivering healthcare to undocumented migrants. Considering that health entails complete physical, mental, and social wellbeing, individuals working in these aspects, whether in governmental or humanitarian settings, were acknowledged as primary healthcare providers.	
**Undocumented migrant: **A person who moves or has moved across an international border and is not authorized to enter or to stay in a state pursuant to the law of that state and to international agreements to which that state is a party. This category encompasses individuals such as rejected or dismissed asylum seekers, undocumented workers, and overstayers.	
**Health advocacy:** Approaches, strategies, practices, acts, endeavors, or initiatives aiming to address disparities and bridge gaps in healthcare systems, with the goal of guaranteeing equal access to affordable, effective, and high-quality healthcare, free from any form of discrimination. While this objective can be achieved at various levels, in this study, we specifically focus on health advocacy at the individual level, particularly among healthcare providers.	

Our inclusive approach considered studies published in the English language, regardless of the varied data collection techniques employed, including interviews, focus group discussions, direct observation, and mixed methods. We did not impose any restrictions on the publication date, recognizing the value of insights gleaned from a diverse temporal landscape. To ensure the comprehensiveness of our review and to avoid the exclusion of any valuable data, studies that included both healthcare providers and undocumented migrants were deemed eligible, provided that the data were disaggregated and presented separately for each cohort in these studies. This approach enabled us to unequivocally distinguish the data pertaining to healthcare providers, which constituted the primary focus of our analysis.

Conversely, qualitative empirical studies with a primary focus that deviated from providing care for undocumented migrants were excluded. This exclusion extended to studies centered on regular migrants or other vulnerable groups unless there was a discernible indication that these groups included undocumented migrants. Our scoping review also excluded various document types, such as quantitative papers, theoretical papers, reviews, commentaries, opinion articles, editorial notes, conference abstracts without accompanying full texts, case reports, guidelines, policy papers, content-analysis papers, books, book chapters, and dissertations.

Utilizing the inclusion and exclusion criteria outlined earlier, we performed a screening of candidate articles based on title, abstract, and full text. Subsequently, collaborative discussions were held to collectively make a final determination regarding article inclusion.

### Search strategy

The research query was transformed into a PICO framework encompassing population, intervention, comparison, and outcome. In comparison to other widely utilized search tools like the SPIDER framework, the PICO framework demonstrates greater sensitivity with an enhanced ability to identify relevant studies, but reduced specificity with a limited ability to exclude irrelevant studies (Methley et al. 2014). Given our objective of conducting a comprehensive search and mitigating the risk of excluding relevant papers, we opted to employ the PICO framework.

The population in focus consisted of undocumented migrants. The intervention was formulated as patient advocacy strategies implemented by healthcare providers while providing healthcare services to undocumented migrants. The comparison was initially left unspecified to maintain flexibility in our analysis and to allow us to explore various potential comparisons during the data analysis phase. Specifically, we considered examining whether different advocacy approaches were adopted by healthcare providers working in different settings, such as governmental versus humanitarian, and among those from different professional backgrounds, such as nurses versus physicians. The outcome was specified as ethical challenges that might emerge within the realm of the intervention. Nevertheless, it is important to note that challenges beyond ethical considerations were also taken into account.

Subsequently, our search strategy was devised in accordance with the PICO framework, and the research question was structured into three primary search blocks: (1) undocumented migrants, (2) patient advocacy, and (3) ethical challenges.^3^ Each search block incorporated a combination of keywords, truncation symbols, and Boolean operators. To identify relevant keywords and terms, FA conducted a preliminary search by exploring the Medical Subject Headings (MeSH) tree structures in selected databases. Within each search block, keywords were combined using the "OR" Boolean operator, while the "AND" operator was employed to combine the three major search blocks.

For the second search block, "patient advocacy," a comprehensive strategy was adopted to encompass keywords and terms such as delivery of healthcare, physician–patient relations, health services accessibility, healthcare disparities, and the right to health. This broad approach was chosen because patient advocacy activities might not be explicitly labeled as such, and such activities could be embedded in the provider-patient relationship without overtly indicating advocacy involvement. We aimed to identify relevant literature that may not explicitly use the term “healthcare providers” but still involve their advocacy efforts in providing care to undocumented migrants. By using this comprehensive search approach, we ensured that our review included diverse perspectives of healthcare providers in various settings and roles.

Following the identification of relevant keywords and terms, FA systematically explored four databases: PubMed/Medline, Embase, Cinahl, and Cochrane Library. The selection of the four databases was based on their relevance to our scoping review and the need to ensure diverse coverage of the qualitative literature.

**Fig. 1:**
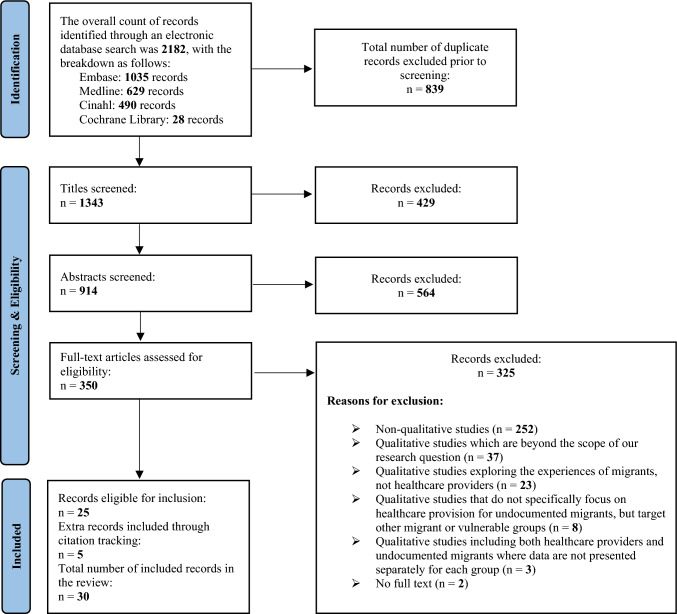
PRISMA flow chart demonstrating the process of identifying relevant articles across four electronic databases

The primary search, executed on March 12, 2023, involved utilizing subject headings specific to each database. The ultimate search strategy and its outcomes underwent collaborative review with co-authors. See Supplement 1 and Supplement 2 for full details. Upon consensus, FA integrated search findings from distinct databases and transferred them to a reference management tool (Endnote). Subsequently, duplicates were eliminated before proceeding to title, abstract, and full-text screening, which was supplemented by the snowball search method. To keep our scoping review recent, we performed a second search on May 20, 2024, using the same search strategy across the same four databases (Supplement 1, Table 5). Additional records were transferred to Endnote, and the same search procedure was repeated. The search procedure conformed to the overarching framework depicted in the PRISMA flow diagram (Fig. 1).

### Data extraction and analysis

We developed a data extraction form to fulfill the objectives of our scoping review. Please see Supplement 3. From all the included studies, we extracted the following information: complete references, publication year and location, study objectives, study design and methods employed, service delivery mode (e.g., governmental or humanitarian), healthcare field or discipline for interviewed providers, total number of healthcare providers in each study, their classification by profession, and ultimately, the results encompassing advocacy approaches and related ethical challenges.

Our analysis drew inspiration from Graneheim and Lundman's approach for qualitative content analysis, particularly their delineation of "manifest" and "latent" content analysis (Graneheim and Lundman 2004). The inductive approach, a manifest content analysis, was employed to discern advocacy approaches or strategies. These were perceived as the most conspicuous elements in the texts of the included papers, as they were clearly stated and easily recognizable.

In contrast, a deductive approach, utilizing latent content analysis, was applied to uncover ethical challenges not always overtly mentioned in the texts. This necessitated a deeper interpretation of the underlying meanings and contexts and a higher level of abstraction of the reported experiences and actions of healthcare providers. For instance, we analyzed the contexts and implications of the advocacy strategies and the challenges encountered by healthcare providers, even when these challenges were not explicitly labeled as "ethical challenges" within the included papers.

To elucidate, the ethical challenges were identified through a meticulous review of the contexts, actions, and experiences described in the included papers. By interpreting these elements, we inferred the ethical dilemmas and issues that healthcare providers might encounter in their advocacy roles. This process involved analyzing the situations and decisions described in the articles and considering their ethical implications, even if they were not directly stated. This additional layer of analysis enabled us to identify and interpret the ethical challenges encountered by healthcare providers in their advocacy efforts.

Our analytical approach considered the results section in the included papers, quotations from interviews, and notes from direct observations as the units of analysis. No constraints were imposed on the meaning units (content or coding units), encompassing words, sentences, and paragraphs within these units. The analytical process involved condensing (shortening) isolated meaning units, followed by aggregation (grouping together under higher order headings). Through condensation and aggregation, we derived codes, categories, and themes.

Based on our analysis, there were:

- A single overarching theme: Employed advocacy approaches by healthcare providers and their correlated ethical challenges
- Two primary categories: (1) advocacy approaches; and (2) ethical challenges
- A total of 30 sub-themes (condensed meaning units): comprising 14 advocacy approaches and 16 ethical challenges
- The advocacy approaches were further categorized into four major groups: (1) voluntary care; (2) understanding and empathy; (3) personal growth and awareness; and (4) systemic health advocacy

We refrained from implementing additional sub-categorization for the recognized advocacy approaches. This decision stemmed from thorough consideration and discussion, as we concluded that suggested sub-categories would lack external heterogeneity (data fitting into more than one sub-category) and would not substantially contribute to an enhanced descriptive level of the content. While there were opportunities for sub-categorizing ethical challenges, we chose to leave them uncategorized. This decision was made to allow the presentation and discussion of these challenges in correlation with advocacy approaches, aligning with our intention to identify and explore these challenges within the context of advocacy approaches.

In ensuring the trustworthiness of our research findings, we meticulously adhered to Graneheim and Lundman's guidelines for credibility, dependability, and transferability. Our findings were considered credible because the amount of generated data was sufficiently rich to address the research question. The healthcare providers in the included studies came from diverse backgrounds, health fields, and professions, contributing to a richer variation of the adopted advocacy approaches.

In our scoping review, we analyzed 30 qualitative studies that fulfilled our inclusion criteria. Throughout the data analysis, we consistently checked for thematic saturation by assessing the themes and sub-themes identified in the selected studies and observing when further data started to repeat existing findings without adding new information. After analyzing 21 studies, we reached a point where no new themes or insights were emerging from the data, demonstrating data richness.

Regarding the appropriateness of our data collection and analysis methods, we ensured that the meaning units—segments of text representing meaningful information—were carefully defined. Meaning units could vary from individual words to entire paragraphs, but our selection was guided by the principle of capturing meaningful information relevant to our research question.

We recognized that words, being small meaning units, could sometimes provide precise insights, whereas paragraphs, as broader units, could convey more context, depth, and capture more complex ideas. Despite this variability, we ensured that our chosen meaning units were neither too broad, which could lead to superficial analysis, nor too small, which could result in fragmented and meaningless data. Achieving this balance relied on our judgment to capture the essence of the reported experiences without sacrificing context or depth. Additionally, we held multiple review sessions to ensure inter-coder reliability. Any discrepancies were discussed and resolved through consensus, minimizing subjectivity and bias in the selection process.

To effectively interpret words as smaller meaning units, we considered several factors: the context in which the words were used, the frequency of the words (as this may highlight important themes), their salience by identifying words that stand out due to their emotional or descriptive power (such as "fulfilling" and "empowerment"), and co-occurrence patterns by examining how words frequently appear together (for example, the co-occurrence of "moral" with "obligation" and "duty" with "volunteer").

In interpreting paragraphs as meaning units, we treated each paragraph as a cohesive unit encapsulating a singular idea or theme (e.g., an ethical dilemma associated with an advocacy practice). We also undertook a contextual interpretation within the broader textual framework, accounting for contextual nuances such as sentiment.

To achieve dependability, we maintained consistency throughout the data collection process. Our research team met regularly to discuss the degree of change in the collected data over time and reached agreements regarding similarities and differences in content. Finally, we believe that our findings can be transferable and applicable to other healthcare providers in similar contexts. However, it is crucial to carefully consider other contextual differences. To enhance transferability, we also included appropriate quotations from the included papers to present our findings.

### Use of large language models

Any use of AI and AI-assisted technologies was conducted ethically and solely for language purposes to improve the manuscript's readability. Specifically, AI was employed for grammar and spell-checking, checking sentence formation, and suggesting minor revisions to ensure the clarity of our writing. AI was not used for generating any content or drawing scientific conclusions.

## Results

### Study characteristics

During our systematic literature search, we identified 1343 records after eliminating duplicates. Among these, 25 records met the eligibility criteria outlined in our inclusion and exclusion criteria (Biswas et al. 2011; Jensen et al. 2011; Dauvrin et al. 2012; Holmes 2012; Marrow 2012; Straßmayr et al. 2012; Tiedje and Plevak 2014; Teunissen et al. 2015; Sandblom and Mangrio 2017; Cervantes et al. 2018; Armin 2019; Bianchi et al. 2019; Fabi and Taylor 2019; Doshi et al. 2020; Granero-Molina et al. 2021, 2022; Hoekstra 2021; Lafaut 2021; van Midde et al. 2021; Saadi et al. 2021; Kvamme and Voldner 2022; Vanobberghen et al. 2022; Mladovsky 2023; Jiménez-Lasserrotte et al. 2023; Gely et al. 2023). Additionally, an extra five records were discovered through citation tracking (Castañeda 2011; Willen 2011; Gullberg and Wihlborg 2014; López-Domene et al. 2019; Piccoli and Perna 2024). Consequently, a total number of 30 records was included in our scoping review. Refer to Fig. 1 for more details. The 30 studies incorporated into our scoping review span the publication years 2011 to 2024. Among these, 27 studies were carried out in ten countries, spanning three continents—North America, Europe, and Asia. Specifically, eleven studies were conducted in the USA (Holmes 2012; Marrow 2012; Tiedje and Plevak 2014; Cervantes et al. 2018; Armin 2019; Bianchi et al. 2019; Fabi and Taylor 2019; Doshi et al. 2020; Hoekstra 2021; Saadi et al. 2021; Gely et al. 2023), four each in Spain (López-Domene et al. 2019; Granero-Molina et al. 2021, 2022; Jiménez-Lasserrotte et al. 2023), two each in Belgium (Lafaut 2021; Vanobberghen et al. 2022), Denmark (Biswas et al. 2011; Jensen et al. 2011), the Netherlands (Teunissen et al. 2015; van Midde et al. 2021), and Sweden (Gullberg and Wihlborg 2014; Sandblom and Mangrio 2017), and one each in England (Mladovsky 2023), Germany (Castañeda 2011), Israel (Willen 2011), and Norway (Kvamme and Voldner 2022). The remaining three studies out of the included 30 were multicentered and conducted across multiple European countries (Dauvrin et al. 2012; Straßmayr et al. 2012; Piccoli and Perna 2024). Additional information regarding the characteristics of the included studies can be found in Supplement 4.

**Fig. 2:**
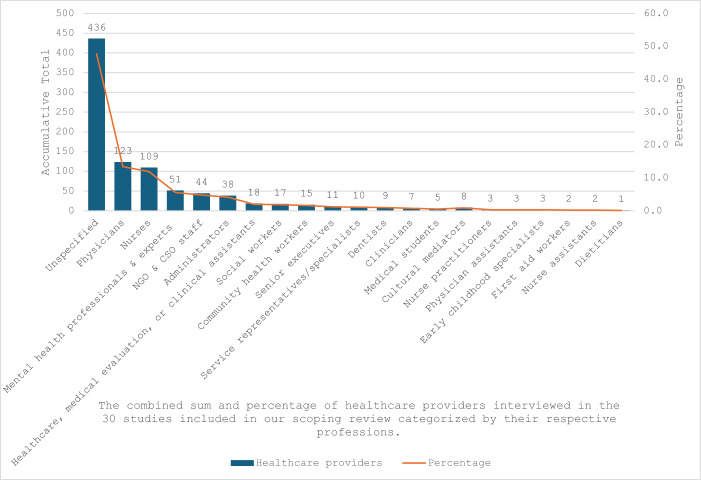
Classification of healthcare providers (accumulative total = 915) based on their professions

As illustrated in Fig. 2, our scoping review encompassed 30 studies, revealing a cumulative total of 915 healthcare providers who were interviewed. The professions of 436 (47.7%) healthcare providers were not explicitly delineated. Among the specified roles, 123 (13.4%) were identified as physicians, 109 (11.9%) as nurses, and 51 (5.6%) as mental health professionals and experts. The remaining 196 (21.4%) healthcare providers came from various professional backgrounds.

As elucidated in Supplement 4, the studies we included exhibited varied sample sizes, ranging from 7 to 240 participants. In 23 studies, the sample size exclusively consisted of healthcare providers, whereas in the remaining seven studies (Biswas et al. 2011; Castañeda 2011; Willen 2011; Holmes 2012; van Midde et al. 2021; López-Domene et al. 2019; Gely et al. 2023), the sample size included both healthcare providers and undocumented migrants. Among the 30 studies considered, five exclusively involved nurses (Biswas et al. 2011; Granero-Molina et al. 2022; Gullberg and Wihlborg 2014; Kvamme and Voldner 2022; Sandblom and Mangrio 2017), while three focused solely on physicians (Granero-Molina et al. 2021; Jensen et al. 2011; Teunissen et al. 2015). The modes of service delivery exhibited variability across the studies we included. Specifically, in twelve studies, healthcare providers operated within the private sector (non-governmental or humanitarian), while in ten studies, they were situated in the public sector (formal or governmental). Additionally, seven studies indicated a mode of service delivery that encompassed both sectors, and in one study, the health sector was not specified. Regarding the methods employed in the incorporated studies, eleven utilized semi-structured interviews. Nine studies employed ethnographic fieldwork, which encompassed both direct observation and interviews. Four studies combined in-depth and semi-structured interviews, while three others used only in-depth interviews. Additionally, one study conducted focus group discussions, another combined focus group discussions and in-depth interviews, and one more combined focus group discussions with semi-structured interviews.

**Fig. 3:**
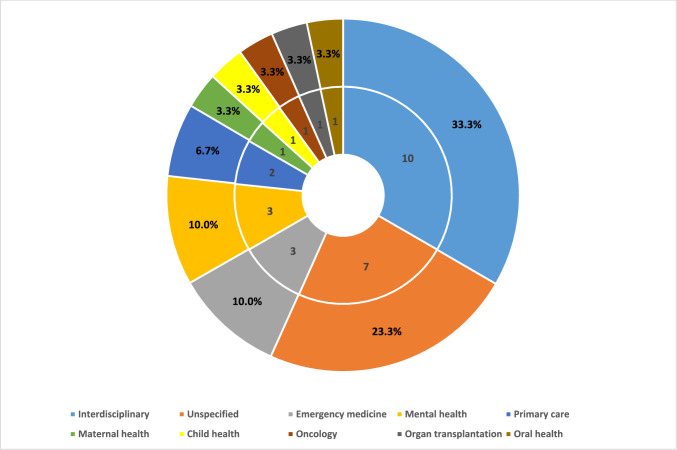
The frequency and proportionate breakdown of the main health fields across the 30 studies included

Figure 3 demonstrates the distribution of the primary health fields among healthcare providers in the included studies. Out of the 30 studies, ten featured an interdisciplinary health field, while seven did not specify any particular field. The remaining studies covered various health fields, such as emergency medicine, mental health, primary care, child health, maternal health, oncology, organ transplantation, and oral health.

### Main findings

Following our analysis, we identified fourteen advocacy approaches and sixteen interconnected ethical challenges. The advocacy approaches were categorized into four groups: Voluntary care, understanding and empathy, personal growth and awareness, and systemic health advocacy. There were no significant differences in advocacy approaches based on healthcare setting or profession; only minor differences were observed, which did not necessitate specific emphasis.

Certain advocacy approaches were correlated with more than one ethical challenge (approaches number 1, 11, 12, and 14), while others lacked such associations (approaches number 6, 7, 9, 10, and 13). When we refer to ethical challenges, we are specifically addressing those that became apparent during our analysis in the context of the implemented advocacy strategies. For a comprehensive overview of the advocacy approaches adopted by healthcare providers, the conceptualization of each advocacy approach, and the corresponding ethical challenges, please find Table 1.

**Table 1:** Overview of advocacy approaches adopted by healthcare providers in the studies included in our scoping review, along with the interrelated ethical considerations with each individual approach

Advocacy approach	Concept	Emerged ethical considerations and other related issues
*Voluntary care*		
*The first approach:* To voluntarily engage in providing care	This advocacy approach highlights the significance of healthcare providers voluntarily contributing their skills and efforts, motivated by compassion and an understanding of the unique healthcare challenges faced by undocumented migrants. These voluntary actions, driven primarily by altruism, demonstrate a commitment that extends beyond professional duty	Striking a balance between the eagerness to assist and professional competence Ensuring that the volunteering efforts are suitable and not risky
*Understanding and empathy*		
*The second approach:* To attempt to grasp undocumented migrants’ experiences	This advocacy approach involves a comprehensive exploration of the life stories, experiences, cultural backgrounds, challenges, and injustices faced by undocumented migrants. It emphasizes the importance of personal connections to understand their realities beyond traditional stereotypes, enabling healthcare providers to deliver culturally sensitive and tailored care that meets their specific health needs	Ensuring cultural sensitivity and steering clear of stereotyping
*The third approach:* To gain a heightened sense of undocumented migrants’ feelings	This advocacy approach emphasizes the importance of empathy and the willingness to step into the emotional world of undocumented migrants to comprehend their fears and despair. By actively cultivating a profound grasp of their feelings, healthcare providers aim to foster a stronger caregiver-patient relationship, acknowledging the migrants' humanity and emotional burdens	The importance of respecting their privacy and establishing trust
*Personal growth and awareness*		
*The fourth approach:* Driving inspiration from undocumented migrants	This advocacy approach highlights the profound impact of interactions with undocumented migrants, inspiring healthcare providers to see their advocacy efforts as more than just professional duties. They draw inspiration from the resilience and strength demonstrated by the undocumented migrant community	Blaming undocumented migrants
*The fifth approach:* A chance of personal growth	This advocacy approach emphasizes the personal and academic benefits that healthcare providers derive from engaging with undocumented migrants, highlighting the richness of the learning experience and the sense of fulfillment that comes from contributing to the well-being of a vulnerable population	The importance of steering clear of a self-centered approach to advocacy
*The sixth approach:* Being present for undocumented migrants	This advocacy approach highlights the significance of "being present" as a potent and supportive gesture. It underscores the idea that healthcare providers, through their active presence and solidarity, contribute to the empowerment and well-being of undocumented migrants, reinforcing the broader goal of building a healthier and more inclusive community	
*The seventh approach:* Delivering additional support beyond medical care	This advocacy approach emphasizes the importance of recognizing and responding to the complex needs of undocumented migrants, including both personal and social aspects. By providing support beyond medical care, healthcare providers contribute to the overall well-being of this population, recognizing the interconnected nature of health and various aspects of life	
*The eighth approach:* Self-awareness of their own position and their own role	This advocacy approach underscores the importance of healthcare providers being conscious of their professional roles and responsibilities in advocating for equitable healthcare. It is not seen as an additional duty but as an integral aspect of their professional responsibilities, requiring providers to be aware of the healthcare entitlements of undocumented migrants	The clash between the personal norms of healthcare providers about care and their institutional roles
*Systemic health advocacy*		
*The nineth approach:* Eliminating obstacles to healthcare access for undocumented migrants	This advocacy approach emphasizes the persistent efforts of healthcare providers to identify, address, and eliminate obstacles to healthcare access for undocumented migrants. It involves not only addressing individual cases but also implementing initiatives to tackle broader challenges in healthcare accessibility for this population	
*The tenth approach:* Using their personal connections to secure access to healthcare	This advocacy approach underscores the significance of healthcare providers utilizing their personal networks to ensure that undocumented migrant patients receive specialized care. It reflects a commitment to going beyond conventional channels to meet the diverse needs of this population	
*The eleventh approach:* To push their limits	This advocacy approach highlights the proactive efforts of healthcare providers to go above and beyond their regular routines. It involves healthcare providers demonstrating the ability to push their limits to ensure comprehensive care for undocumented migrant patients	Establishing clear personal boundaries Ensuring the well-being of the advocate Avoiding any compromise in the quality of care
*The twelfth approach:* Providing care whenever they can	This advocacy approach highlights the resourcefulness and adaptability of healthcare providers who navigate complex healthcare systems to ensure that undocumented migrants receive the necessary medical attention, often through strategic utilization of existing policies and frameworks	The burden on the healthcare system The need for transparency and accountability The challenges of regulatory compliance along with the potential for legal repercussions
*The thirteenth approach:* Providing affordable healthcare	This advocacy approach underscores the importance of collective action and collaboration among healthcare providers to create affordable healthcare networks. By doing so, they address financial barriers, ensuring that undocumented migrants have access to diverse medical services	
*The fourteenth approach:* Offering care to undocumented migrants when there is no reimbursement	This advocacy approach underscores the commitment of healthcare providers to provide essential care to undocumented migrants, even in the absence of reimbursement. While addressing immediate healthcare needs, it also raises critical questions about fairness, budget considerations, and the broader responsibility for covering treatment expenses for this population	The economic burden on healthcare providers Maintaining fairness principles while avoiding favoritism in healthcare Having confidence in the intentions of undocumented migrants seeking free treatments and services

Certain advocacy approaches were correlated with more than one ethical challenge (approaches number 1, 11, 12, and 14), while others lacked such associations (approaches number 6, 7, 9, 10, and 13)

### Voluntary care

#### The first approach: to voluntarily engage in providing care

This advocacy approach emerged as a theme in the following studies: (Vanobberghen et al. 2022; Granero-Molina et al. 2021, 2022; Teunissen et al. 2015; Sandblom and Mangrio 2017; Tiedje and Plevak 2014; Hoekstra 2021; Armin 2019; Castañeda 2011; Jiménez-Lasserrotte et al. 2023; Piccoli and Perna 2024). Numerous healthcare providers, as mentioned in various papers (Granero-Molina et al. 2021; Sandblom and Mangrio 2017; Tiedje and Plevak 2014; Hoekstra 2021; Castañeda 2011), described volunteering to provide healthcare for undocumented migrants as the most fitting and essential response to meet the healthcare needs of this patient group. This act is underscored as a pivotal and necessary initiative in order to address and fulfill the unique health challenges faced by undocumented migrants. In a study conducted to assess experiences of healthcare providers during medical monitoring of a hunger strike in Belgium (Vanobberghen et al. 2022, p. 4), one of the participated nurses expressed “*We are really getting out of the normal routine, and we are not even forced to do it*”. This emphasizes that the decision to volunteer is a voluntary and compassionate choice rather than an obligation. It implies a sense of willingness and dedication to assisting those in need, even though it requires a departure from their typical routine. In line with findings of (Granero-Molina et al. 2022, p. 74), the choice to volunteer in delivering healthcare to undocumented migrants arriving in Spain via small boats was genuine and stemmed from the volunteers' altruistic motivation to assist. The collaborative approach was also recognized as an essential aspect of volunteering.

Healthcare providers used diverse expressions to convey their experiences, such as "*being a volunteer makes me feel important*" (Granero-Molina et al. 2021, p. 3). Others perceived volunteering not only as a fundamental human duty but also as a responsibility, especially when others neglect their responsibility to care for undocumented migrants and address their healthcare needs (Sandblom and Mangrio 2017, p. 5). As an illustration, one of the physicians who provided care to undocumented migrants in Berlin, Germany, saw her volunteering experience as akin to volunteering as a doctor in a third-world country.^4^ In both situations, healthcare providers felt compelled to volunteer and respond with humanitarian efforts, showcasing a common motivation to address healthcare needs in various challenging contexts. Providing care to undocumented migrants was recognized as one of these demanding situations (Castañeda 2011, p. 5). While healthcare providers may be willing to volunteer, various factors could potentially diminish their inclination to do so. Granero-Molina et al. highlighted some of these factors, including family commitments, limited awareness of volunteering opportunities, and a lack of options for retired healthcare providers (Granero-Molina et al. 2021, p. 3).

Concerning the first advocacy approach, our analysis reveals two key ethical challenges: striking a balance between the eagerness to assist and professional competence, and ensuring that the volunteering efforts are suitable and not risky. These challenges are evident in the following quote from one of the interviewed physicians:

“The one that always sticks out in my mind was the young man with testicular cancer. We were able to do an outpatient operation and sent him home the same day. He stayed with his sister and slept in her kitchen. Can you imagine! I remember going over there to pull the stiches, right there on the kitchen table. It certainly was not ideal circumstances with heightened danger of infection after a surgery like that. … And then we had to arrange for the chemotherapy. At the time, the standard was three rounds of treatment, and that required a hospital stay. So, I spent a lot of time consulting with colleagues, calling people and seeing if they could recommend anything. Finally, someone suggested giving only one round of treatment. That sounds like we were providing standard care, but as it turns out, that is exactly what they do in [another European country], just the one round of treatment. And they seem to have good results. So that is what we had to do, one round of treatment, and the patient did wonderfully. I didn’t know anything about the law at the time I treated him—just that I would not be paid and that it was not exactly legal” (Castañeda 2011, p. 5).

In our interpretation, this quote vividly illustrates the lengths to which healthcare providers go to ensure their patients receive necessary care, often navigating significant legal and ethical challenges. The physician’s narrative underscores the complexity and dedication involved in providing voluntary care under less-than-ideal circumstances. The physician had to perform a procedure in a non-clinical setting (the patient's kitchen), which increased the risk of infection. This scenario highlights the ethical challenge of ensuring safety while engaging in risky volunteer efforts to respond to the urgent needs of undocumented migrants. Furthermore, the physician's proactive approach in seeking alternative treatment options to provide standard care and consulting with colleagues demonstrates a desire to balance eagerness to assist with professional competence. While the patient had a positive outcome, the process involved making some ethical and legal compromises. The challenge lies in justifying these compromises given the successful result and considering whether similar decisions should be made in future cases.

### Understanding and empathy

#### The second approach: To attempt to grasp undocumented migrants’ experiences

Our analysis revealed a prominent theme that drew our attention as a noteworthy advocacy approach—delving into the experiences of undocumented migrants (Vanobberghen et al. 2022; Granero-Molina et al. 2021; Granero-Molina et al. 2022; Sandblom and Mangrio 2017; Bianchi et al. 2019; Tiedje and Plevak 2014; Saadi et al. 2021; Doshi et al. 2020; Hoekstra 2021; López-Domene et al. 2019; Willen 2011; Jiménez-Lasserrotte et al. 2023). Healthcare providers elaborated that this approach heightened their awareness of the health needs of undocumented migrants by uncovering the life stories underlying their health issues (Granero-Molina et al. 2021, p. 3). This method served as a means to explore both the patient's world and their own, gaining insights into the reality of undocumented migrants, their fears, and health conditions (Granero-Molina et al. 2021, p. 3). They conveyed that advocating for the health rights of undocumented migrants exposed them to a wholly new experience (Vanobberghen et al. 2022, p. 4), driven by a steadfast belief that these individuals face unjust demands, and hosting countries are falling short in ensuring their health rights (Sandblom and Mangrio 2017, p. 4).

Additionally, some healthcare providers championed the rights of undocumented migrants due to their personal experiences by being either immigrants themselves or the offspring of immigrants (Saadi et al. 2021, p. 3077). One provider emphasized that the immigrant background of his family significantly influenced his advocacy choices (Willen 2011, p. 318). Others empathized by imagining themselves in the shoes of undocumented migrants, expressing a desire for someone to assist and advocate for their rights (Bianchi et al. 2019, p. 826). Consequently, these healthcare providers endeavored to support undocumented migrants beyond healthcare, recognizing the multifaceted challenges they encounter (Vanobberghen et al. 2022; Granero-Molina et al. 2021, 2022).

Nevertheless, a healthcare provider highlighted that advocacy efforts could be as simple as allowing undocumented migrants to share their stories and be heard, asserting that, in many instances, this is all they truly need (Doshi et al. 2020, p. 12). Understanding the experiences and cultural backgrounds of undocumented migrants is crucial not only for advocating for their health rights but also for delivering more effective, culturally sensitive care tailored to their specific health needs (Vanobberghen et al. 2022; López-Domene et al. 2019). This advocacy approach was particularly significant in the context of undocumented migrant women arriving in Spain via small boats (López-Domene et al. 2019). A substantial number of these women fell victim to trafficking networks that exploited their children and families as leverage to exert control. Many were underage girls coerced by traffickers into falsely claiming legal age to subject them to sexual exploitation. These women and girls had their sexual lives manipulated by trafficking networks, leading to instances of sexual assault. Some were even compelled to undergo illegal abortions in advanced pregnancies. Additionally, some women were given children who were not biologically theirs, underscoring the importance of examining the child-mother relationship (López-Domene et al. 2019).

Healthcare providers who cared for these undocumented migrant women upon arrival brought attention to these experiences (López-Domene et al. 2019). Failure on the part of these providers to comprehend these women's experiences and explore their migration journeys would hinder their ability to deliver the necessary care and support. Consequently, they would be unable to fulfill the needs of these women and advocate for their rightful health rights.

In the scope of the second advocacy approach, our analysis of the following papers (Granero-Molina et al. 2021, 2022; Tiedje and Plevak 2014; Doshi et al. 2020; Saadi et al. 2021; Willen 2011) revealed a specific ethical challenge: ensuring cultural sensitivity and steering clear of stereotyping. Healthcare providers should acknowledge and respect the diverse cultural backgrounds of undocumented migrants, refraining from making sweeping assumptions about this group. This entails comprehending their beliefs, values, and communication styles, while recognizing that each undocumented migrant possesses individual and distinctive experiences.

#### The third approach: to gain a heightened sense of undocumented migrants’ feelings

This advocacy approach was utilized by healthcare providers in the following papers: (Vanobberghen et al. 2022; Granero-Molina et al. 2021; Sandblom and Mangrio 2017; Jiménez-Lasserrotte et al. 2023). In the pursuit of asserting their rights, undocumented migrants employ practices that embody resistance, serving as poignant protests against the injustices they endure. These acts, though possibly perceived as extreme and perplexing by healthcare providers, carry profound significance.

In 2014, a cohort of 200 undocumented migrants, primarily families with children, initiated a hunger strike in Brussels (Belgium). Intriguingly, 18 compassionate healthcare providers, comprising young health professionals and medical students, volunteered to oversee the medical aspects of this protest. Beyond their altruistic motives, their participation stemmed from a genuine curiosity about the living conditions and motivations driving the migrants to undertake such a drastic measure (Vanobberghen et al. 2022). This convergence of healthcare providers and undocumented migrants led to a complex dynamic. Many of the providers found themselves navigating uncharted territory, providing care in conditions they had never encountered before. The internal conflict they grappled with regarding their roles as caregivers added an additional layer of complexity. Some providers perceived the actions of undocumented migrants as extreme and confusing, questioning the reasoning behind putting their health at risk. These perceptions created an obstacle to delivering effective care. In response, one strategic approach they embraced involved immersing themselves in the emotions of undocumented migrants, striving to comprehend their despair firsthand, without passing judgment. This unique strategy aimed to cultivate a heightened sense of empathy among healthcare providers, fostering a connection that transcends the conventional caregiver-patient relationship.

Other healthcare providers adopted the same approach (Granero-Molina et al. 2021; Sandblom and Mangrio 2017). Their perspective extended beyond the clinical realm, emphasizing that addressing the healthcare needs of these individuals is tantamount to acknowledging their existence (Sandblom and Mangrio 2017, p. 5). It surpasses the confines of routine clinical practice, transforming into a deep grasping of their emotions and the burdens they bear (Granero-Molina et al. 2021, p. 3). We find that by deeply comprehending the feelings of undocumented migrants and recognizing the emotional impact of their circumstances on their health, healthcare providers can develop more targeted and holistic care, ensuring that the health needs of undocumented migrants are adequately addressed.

To better comprehend the emotions of undocumented migrants, our analysis of two papers revealed a significant ethical challenge: the importance of respecting their privacy and establishing trust (Sandblom and Mangrio 2017; Vanobberghen et al. 2022). Given that undocumented migrants often live in fear, establishing a connection with them can be challenging. Therefore, healthcare providers must actively work to earn their trust.

### Personal growth and awareness

#### The fourth approach: Driving inspiration from undocumented migrants

According to our analysis, this advocacy approach emerged as a recurring theme in the following papers: (Vanobberghen et al. 2022; Kvamme and Voldner 2022; Sandblom and Mangrio 2017; Holmes 2012; Jiménez-Lasserrotte et al. 2023). Drawing inspiration from undocumented migrants, certain healthcare providers found profound motivation in aligning their commitment to care with the resilience observed in the undocumented migrant community (Vanobberghen et al. 2022, p. 5). Their encounters with providing care were not just routine tasks but transformative experiences, as if unveiling a deeper understanding of humanity (Sandblom and Mangrio 2017, p. 5). One healthcare provider marveled, expressing, "*I was amazed by how they keep going and seem happy and content despite their difficult lots in life*" (Holmes 2012, p. 877). Another provider went beyond conventional praise, labeling undocumented migrants as "*the best and the bravest*", elevating their essence to a symbol of courage (Holmes 2012, p. 877).

In a Norwegian study conducted by Kvamme et al., public health nurses, after engaging with undocumented migrant mothers, depicted them as embodiments of strength and resourcefulness. The nurses were captivated by the mothers' ability to nurture their children in the face of an exceedingly uncertain life (Kvamme and Voldner 2022, p. 288). One nurse shared her astonishment, stating, "*It is really amazing how close and caring they are, and calm. They manage to relate to the child when everything else is really uncertain*" (Kvamme and Voldner 2022, p. 288). This portrayal transcends the ordinary, painting a vivid picture of undocumented migrants as resilient, courageous, and deeply nurturing individuals.

While some healthcare providers find inspiration in the resilience and determination of undocumented migrants pursuing a better life, others cast blame upon them, holding them partly responsible for their compromised health conditions (Holmes 2012). Based on our analysis, attributing blame to undocumented migrants emerged as an ethical challenge, as it has the potential to affect the relationship between healthcare providers and patients, as well as the quality of care delivered. This is because access to health services may be viewed more as a privilege than an inherent right.

#### The fifth approach: a chance of personal growth

For certain healthcare providers, providing care to undocumented migrants represents more than just a professional obligation—it serves as a fertile ground for personal and academic enrichment (Granero-Molina et al. 2021; Tiedje and Plevak 2014; Hoekstra 2021; Jiménez-Lasserrotte et al. 2023). This rings particularly true for young healthcare providers seeking to gain valuable experience working with underserved communities (Hoekstra 2021), and for specialized professionals aiming to explore the holistic aspects of medicine (Tiedje and Plevak 2014).

One physician articulated this sentiment, stating, “*I have the desire to help others. Volunteering here helps me to go back to the origin of my profession [medicine]*”. Despite spending the majority of his time in the operating room at a prominent research hospital, this physician sees providing care to undocumented migrants as a means to break free from his routine (Tiedje and Plevak 2014). Others shared their experiences as enriching learning opportunities, providing exposure to uncommon medical conditions (Tiedje and Plevak 2014). For some, the act of delivering healthcare to this population brings a sense of peace to their souls, offering solace in knowing they've contributed to someone else's well-being (Granero-Molina et al. 2021).

In the framework of this advocacy approach, our analysis of one paper (Hoekstra 2021) identified an ethical challenge: the importance of steering clear of a self-centered approach to advocacy. This challenge underscores the need to ensure that personal growth is in harmony with the overarching objective of aiding undocumented migrants, without seeking gratitude or other rewards in return.

#### The sixth approach: being present for undocumented migrants

In standing alongside undocumented migrants and being present for them (Vanobberghen et al. 2022; Granero-Molina et al. 2021; Kvamme and Voldner 2022; Sandblom and Mangrio 2017; Bianchi et al. 2019; Tiedje and Plevak 2014; Saadi et al. 2021; Armin 2019; Castañeda 2011; Willen 2011; Gely et al. 2023; Piccoli and Perna 2024), the gesture transcends the mere act of volunteering or extending practical aid. It can be as simple as expressing solidarity in various ways, such as offering medical advice (Vanobberghen et al. 2022, p. 5), or making statements on the best interests of the child during family or parental deportation cases (Kvamme and Voldner 2022, p. 290). Even if these statements lack the power to halt deportations, they serve as a gesture of support for undocumented migrants and acknowledgment of their challenges. One healthcare provider viewed being present for undocumented migrants as her contribution to the community, describing it as an integral part of her identity and professional duty (Tiedje and Plevak 2014, p. 365). Another healthcare provider emphasized that being present is not an act of charity but rather stems from a commitment to building a healthier community (Tiedje and Plevak 2014, p. 364). Additionally, being present for undocumented migrants is viewed as a means of empowering them to voice their concerns (Saadi et al. 2021, p. 3075).

#### The seventh approach: delivering additional support beyond medical care

Healthcare providers, as indicated in several studies (Straßmayr et al. 2012; Kvamme and Voldner 2022; Saadi et al. 2021; Doshi et al. 2020; Armin 2019), demonstrated their advocacy for undocumented migrants by offering additional support beyond medical care. For instance, in a study led by Kvamme et al., public health nurses supported the children of undocumented migrants by supplying them with clothing and essential equipment (Kvamme and Voldner 2022, p. 290). Some healthcare providers believed that their responsibilities extended beyond the clinic setting and their designated roles (Saadi et al. 2021, p. 3077). This included assisting undocumented migrants in translating formal government communication letters (Doshi et al. 2020, p. 13). In a study conducted by Armin, a healthcare provider went beyond merely providing information to breast cancer patients among undocumented migrants. She also supported them by accompanying them to mammography appointments and offering language interpretation services (Armin 2019, p. 6).

### The eighth approach: self-awareness of their own position and their own role

This scoping review delineates diverse advocacy strategies, encompassing actions like volunteering and providing supplementary aid. However, the advocacy approach highlighted here, involving healthcare providers' self-awareness of their position and role (Lafaut 2021; Fabi and Taylor 2019; Saadi et al. 2021; Gullberg and Wihlborg 2014; Armin 2019; Piccoli and Perna 2024), is not presented as an extra duty. Instead, it is underscored as an integral aspect of their professional responsibilities as caregivers. This includes ensuring equitable care for all patients, including marginalized individuals, and addressing their health needs fully.

Healthcare providers can express self-awareness in various ways, including recognizing their duty in rationing healthcare services within the framework of exclusive health policies and addressing administrative invisibility for undocumented migrants (Armin 2019, p. 6). This involves informing them about their rights and assisting them in navigating the healthcare system (Fabi and Taylor 2019; Gullberg and Wihlborg 2014). However, for healthcare providers to accomplish this, they must expand their comprehension of the health rights specific to undocumented migrants, familiarize themselves with relevant health policies, acts, and legislation applicable to this patient group, and identify the available healthcare services and initiatives designated for them (Armin 2019).

Several healthcare providers operating in humanitarian settings underscored the significance of self-awareness regarding their professional roles. This awareness goes beyond providing care exclusively through non-governmental organizations; it also involves integrating undocumented migrants into public healthcare systems. They see their roles as caregivers as secondary or temporary, serving the purpose of bridging gaps in accessibility to specific healthcare services (Lafaut 2021, p. 6).

In the realm of this advocacy approach, a notable challenge emerged, highlighting the clash between the personal norms of healthcare providers about care and their institutional roles. Consequently, they needed to devise inventive strategies to deliver suitable care while navigating the limitations imposed by their roles and the dynamics of their institutions (Lafaut 2021, p. 7).

### Systemic health advocacy

#### The ninth approach: eliminating obstacles to healthcare access for undocumented migrants

Eliminating obstacles to healthcare access emerged as an advocacy approach in nine studies: (Fabi and Taylor 2019; Dauvrin et al. 2012; Kvamme and Voldner 2022; van Midde et al. 2021; Tiedje and Plevak 2014; Holmes 2012; Armin 2019; Castañeda 2011; Gely et al. 2023). Numerous obstacles hinder healthcare accessibility for undocumented migrants, operating at individual, institutional, and societal levels. For instance, at the individual level, undocumented migrants face heightened risks of occupational hazards due to poor working conditions and the absence of work insurance. A specific case highlighted in a study by Holmes involved an undocumented migrant diagnosed with a life-threatening lung infection (Valley Fever), necessitating lifelong anti-fungal suppression medications costing 1000 USD per month. The patient lacked the financial means and health insurance to cover the expense. The dedicated physician overseeing the case had to explore special programs and invest significant time and effort to secure the necessary medications for the patient. This demanding process had to be repeated monthly, reflecting the ongoing challenges faced by healthcare providers in such situations (Holmes 2012, p. 876).

Following a similar approach, other healthcare providers were able to extend support to additional undocumented migrant patients with conditions like cancer (Castañeda 2011; Armin 2019). Some of these providers even established networks to address specific challenges in healthcare accessibility for undocumented migrants, such as oral health (van Midde et al. 2021), and cancer screening (Armin 2019).

An additional challenge underscored in one of the studies was the absence of a permanent address for undocumented migrants. To address this issue and ensure the continuity of care, providers sought flexibility in scheduling follow-up appointments, employing alternative methods such as coordinating consultations on specific dates or maintaining contact through telephone or SMS (Kvamme and Voldner 2022, p. 290).

#### The tenth approach: using their personal connections to secure access to healthcare

In various studies (Fabi and Taylor 2019; Straßmayr et al. 2012; Dauvrin et al. 2012; Jensen et al. 2011; Armin 2019; Castañeda 2011), numerous healthcare providers have successfully secured access to certain healthcare services for their undocumented migrant patients by leveraging personal connections. This approach proved particularly crucial for facilitating specialty care and ensuring access to healthcare services that these patients could not afford. For instance, in a study led by Fabi et al., a midwife shared an instance where she arranged access to an echocardiogram for her undocumented pregnant patient by contacting her personal cardiologist. She expressed, “*This is the sort thing I would do for my daughter*” (Fabi and Taylor 2019, p. 402). The same strategy was employed to obtain access to other services, including mental health and community services (Straßmayr et al. 2012; Armin 2019). A community healthcare worker explained, “*I have a big blinder and I keep adding to it every time I go to health affairs… I have a stuff on domestic violence, clothing banks, child protection services… We need to know about this stuff because we never know what patients are going to ask for*” (Armin 2019, p. 6).

#### The eleventh approach: to push their limits

Several healthcare providers, as indicated in various studies (Vanobberghen et al. 2022; Teunissen et al. 2015; Doshi et al. 2020; Hoekstra 2021; Castañeda 2011; Willen 2011), have demonstrated the ability to step outside their regular routines and push their limits by implementing specific strategies. These include concentrating their consultations with undocumented migrant patients (Teunissen et al. 2015, p. 87), scheduling off-hour appointments (Doshi et al. 2020, p. 13), and conducting home visits for the most vulnerable patients, particularly those with chronic conditions like paralysis (Hoekstra 2021, p. 6).

In conjunction with this advocacy approach, our analysis identified three significant ethical challenges: establishing clear personal boundaries (Willen 2011; Hoekstra 2021; Doshi et al. 2020), ensuring the well-being of the advocate (Vanobberghen et al. 2022; Teunissen et al. 2015), and avoiding any compromise in the quality of care (Willen 2011; Castañeda 2011). To illustrate, a study by Willen highlighted a case where an oncologist administered chemotherapy to a cancer patient in his own kitchen (Willen 2011, p. 312). While the oncologist's advocacy efforts are commendable, it is worth considering that some of the aforementioned ethical issues may be relevant in such cases.

Healthcare providers need to balance their professional duties with their personal lives to prevent burnout. Constantly overextending themselves in providing healthcare to undocumented migrants can lead to emotional and physical exhaustion (Willen 2011; Vanobberghen et al. 2022). Departing from a regular routine can sometimes be challenging. For example, during the medical monitoring of a hunger strike initiated by undocumented migrants, a number of healthcare providers had to prioritize their own well-being. Consequently, they found themselves unable to continue meeting their commitments to these migrants and ultimately made the difficult decision to withdraw (Vanobberghen et al. 2022). Extending care beyond regular hours and traditional healthcare pathways is commendable, as it enhances healthcare accessibility for undocumented migrants. However, this approach can sometimes compromise the quality of care. Despite good intentions, we find that such actions may not always adhere to standard care protocols.

#### The twelfth approach: providing care whenever they can

Numerous healthcare providers, as evidenced by various studies (Fabi and Taylor 2019; Mladovsky 2023; Dauvrin et al. 2012; Jensen et al. 2011; Gullberg and Wihlborg 2014), shared instances where they skillfully were able to get around the healthcare systems and existing policies to ensure accessibility to healthcare services for undocumented migrants.

For instance, in a study conducted in the United States by Fabi et al., a primary healthcare provider in California outlined their ability to obtain medical services under the prenatal policy for pregnant women, even when the policy itself might not fully encompass such services. The provider explained that this strategic approach allowed them to efficiently manage all necessary medical aspects while patients are still insured (Fabi and Taylor 2019, p. 402). Furthermore, other healthcare providers in the same study mentioned leveraging available insurance options during pregnancy to cover medical appointments, treatments, or procedures not explicitly addressed by the policy, although their descriptions of the specific methods employed were somewhat ambiguous (Fabi and Taylor 2019, p. 402).

In a separate study conducted by Mladovsky, certain clinical psychologists successfully adapted and utilized medicalization as a rationale for health coverage, offering undocumented migrants social support to mitigate the adverse impacts of the immigration system in the United Kingdom. This was achieved by reallocating mental care funding from the NHS to facilitate the provision of comprehensive social support. For instance, they aided their patients in accessing better housing by demonstrating to housing authorities that their patients' medical conditions were either caused or exacerbated by their current housing conditions (Mladovsky 2023, p. 4).

Other healthcare providers implemented alternative approaches, including arranging unregistered check-up visits for undocumented migrants (Dauvrin et al. 2012, p. 3), prescribing medications under their own names (Dauvrin et al. 2012, p. 3), and conducting tests using their personal information or a fabricated social number (Jensen et al. 2011, p. 5). Finally, in a Swedish study, one provider shared the scenario of admitting an undocumented migrant man with severe chest pain to the pediatric ward, leveraging the coverage available for health services in that age group. Although the man initially claimed to be 17, his appearance suggested an older age, and he admitted to this a few days after admission. Nevertheless, healthcare providers continued to deliver care to him (Gullberg and Wihlborg 2014, p. 154).

Although healthcare providers harbored positive intentions, our analysis revealed three prominent ethical challenges that emerged as interconnected themes: the burden on the healthcare system (Fabi and Taylor 2019; Dauvrin et al. 2012), the need for transparency and accountability (Dauvrin et al. 2012; Jensen et al. 2011; Gullberg and Wihlborg 2014), and the challenges of regulatory compliance along with the potential for legal repercussions (Jensen et al. 2011; Dauvrin et al. 2012).

While healthcare providers endeavor to address the immediate needs of undocumented migrants, they must also consider the broader repercussions for the healthcare system. Previous measures, such as arranging unregistered check-up visits or conducting tests using fabricated social security numbers (Dauvrin et al. 2012; Jensen et al. 2011), have met some urgent needs. However, we find that such measures could place additional strain on the system. Circumventing established healthcare systems and policies can deplete limited resources, thus affecting service availability for other patients.

Consequently, providers encounter a complex dilemma in balancing resource allocation, ethical principles, and legal compliance. This challenge is encapsulated in the following quote: “*I think everyone should [have] access to healthcare and to insurance. So that frustrates me, that we have to kind of maneuver around the system to get people to where they need to be at times. You get to use some creativity, but … some people—they can even break the law at times, and I am not willing to do that. But I think you can sort of figure that out, that if you cannot get what you want to do, people become creative and sometimes it may open the door to doing things that they probably should not be doing. We do not do that within our clinic, but I have seen it done locally with other providers*” (Fabi and Taylor 2019, p. 404).

#### The thirteenth approach: providing affordable healthcare

This approach became evident in the subsequent studies: (Fabi and Taylor 2019; Armin 2019; Hoekstra 2021). In a U.S. study led by Armin, the focus was on delineating advocacy initiatives among diverse healthcare providers overseeing specialized cancer care, including undocumented individuals excluded from state programs (Armin 2019). The study showcased instances of healthcare providers forming a network of 25–30 physicians who were able to deliver pro bono or discounted cancer treatment (Armin 2019, p. 6). A different network of volunteers with diverse backgrounds collaborated to deliver high-quality services to undocumented migrant patients at a cost of only 200 USD per patient per year (Hoekstra 2021, p. 6). Both networks were established in response to the urgent need for such support systems and in acknowledgment of the health rights of undocumented migrants to receive accessible and equitable healthcare services.

#### The fourteenth approach: offering care to undocumented migrants when there is no reimbursement

Numerous healthcare providers, as outlined in various studies (Straßmayr et al. 2012; Teunissen et al. 2015; Jensen et al. 2011; van Midde et al. 2021; Tiedje and Plevak 2014; Castañeda 2011), have encountered scenarios requiring them to provide care to undocumented migrants without receiving reimbursement. This may involve refraining from charging undocumented migrants (Teunissen et al. 2015; van Midde et al. 2021; Jensen et al. 2011), or covering expenses for medications and certain tests out of their own resources (Straßmayr et al. 2012, p. 7). However, this approach has been perceived as a temporary and constrained solution. Despite healthcare providers foregoing financial charges, undocumented migrants are still faced with the obligation to cover costs associated with medications and medical tests. Consequently, the issue of healthcare service coverage for undocumented migrants has prompted inquiries into fairness, budget allocation, and the responsibility for treatment expenses (Straßmayr et al. 2012; Jensen et al. 2011). Many healthcare providers continue to grapple with unresolved questions in these areas.

Concerning this advocacy approach, our analysis revealed three interrelated ethical challenges: the economic burden on healthcare providers (van Midde et al. 2021; Straßmayr et al. 2012; Jensen et al. 2011), maintaining fairness principles while avoiding favoritism in healthcare (van Midde et al. 2021), and having confidence in the intentions of undocumented migrants seeking free treatments and services (van Midde et al. 2021).

When healthcare providers offer care without receiving reimbursement or cover the costs of medications and tests from their own pockets, they may face economic burdens. This can lead to financial strain on the providers and may not be sustainable in the long term. Additionally, providers bear the responsibility of determining if undocumented migrants genuinely need care and are not misusing free healthcare services. They may also struggle with the ethical challenge of treating undocumented migrants fairly while ensuring fairness towards other patients who might also be financially struggling. This challenge involves balancing the needs of undocumented migrants with those of the broader patient population to ensure that no group is unduly favored or neglected.

For instance, when exploring dentists' perspectives on providing free oral care to undocumented migrants, their treatment decisions were influenced by factors such as a personal connection with the patient, feeling compassion for the patient, trust in the patient's intentions to obtain free treatment, and the patient's expression of gratitude (van Midde et al. 2021). This may lead them to favor some patients over others, which could be unfair for patients who fail to show gratitude or earn the providers' compassion, despite their genuine need for free services.

## Conceptual framework for advocacy approaches

**Fig. 4:**
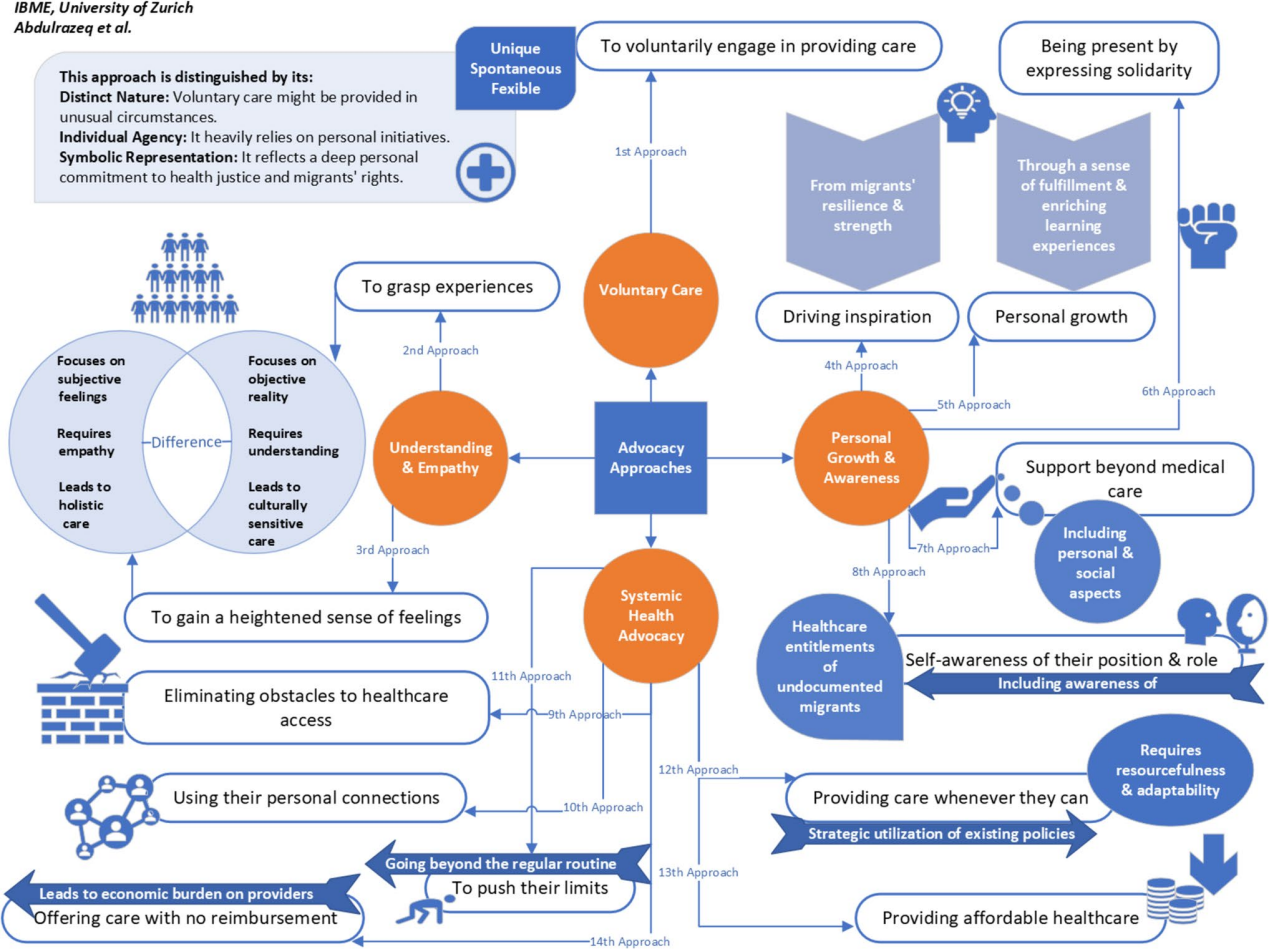
Concept map for categorizing and distinguishing advocacy approaches employed by healthcare providers in delivering care to undocumented migrants

After presenting our main findings, and before moving on to the discussion, we would like to clarify a few points regarding the distinctions and rationale behind our categorization of advocacy approaches (Fig. 4).

***Categorizing the first approach:*** In the context of volunteering, healthcare providers may integrate their voluntary efforts with other advocacy strategies, such as understanding patients' experiences, developing a heightened empathy for their feelings, and offering support beyond medical care. Nonetheless, these advocacy strategies are not limited to volunteering; they can also be implemented by healthcare providers as part of their routine responsibilities in both governmental and private healthcare settings. Therefore, volunteering was categorized separately due to its unique nature, characterized by spontaneity and reliance on personal initiative. Voluntary care is generally more flexible compared to formal healthcare delivery and symbolizes healthcare providers' deep commitment to the health rights of undocumented migrants, reflecting their deliberate choice to volunteer. In contrast, other advocacy strategies may be employed out of necessity, as healthcare providers might find themselves advocating for the health entitlements of undocumented migrants because they are part of their patient population.

***Distinguishing between the second and third approaches:*** In exploring the experiences of undocumented migrants, certain healthcare providers may emotionally distance themselves from these patients as a self-protective measure. Consequently, the second and third advocacy strategies emerged as distinct themes, indicating that healthcare providers can employ these approaches separately without needing to integrate them into a unified approach. This distinction allows for flexibility in their advocacy efforts.

Furthermore, we differentiate between experiences and emotions. Experiences pertain to objective reality: the actual events, situations, and real-life narratives that undocumented migrants encounter. The objectivity of experiences is grounded in factual occurrences, representing the tangible realities faced by undocumented migrants in their daily lives. Understanding these circumstances and specific challenges, including cultural, social, and legal aspects, facilitates the provision of culturally sensitive care. Conversely, emotions pertain to subjective feelings, referring to the personal, internal emotional responses of undocumented migrants. These are subjective because they are based on individual perceptions, encompassing fears, hopes, anxieties, and stress. Addressing emotions necessitates empathy, requiring healthcare providers to connect with the emotional experiences of undocumented migrants on a personal level. This approach leads to holistic care that considers the emotional well-being of the patient, in addition to their physical health.

***Distinguishing between the fourth and fifth approaches:*** By driving their inspiration from undocumented migrants, healthcare providers seek a motivational force to advocate for the healthcare rights of this population. This motivational power is derived from their direct interactions with undocumented migrants, observing their resilience and courage. The fifth approach highlights the profound impact of these interactions on healthcare providers, emphasizing the enriching learning experiences and the sense of fulfillment gained from these engagements.

***Categorizing the sixth approach:*** The approach of “being present for undocumented migrants” was categorized under “personal growth and awareness” because healthcare providers view their presence as essential to their identity and professional responsibility, thereby expressing solidarity with undocumented migrants. Reaching this conclusion requires a heightened level of awareness, ultimately encouraging healthcare providers to engage in reflective practice, gain deeper insights into their roles, and align their values with broader principles of social justice.

***Distinguishing between the sixth, seventh, and eighth approaches:*** Healthcare providers emphasize expressing solidarity with undocumented migrants through their presence, even in small gestures such as offering medical advice. Delivering support that extends beyond medical care includes providing both personal and social assistance. This approach arises from healthcare providers' understanding of the impact that precarious life situations have on the health of undocumented migrants. Lastly, self-awareness involves healthcare providers recognizing their responsibilities within the broader healthcare system. This requires them to be knowledgeable about the healthcare entitlements of undocumented migrants, as well as existing policies, programs, and initiatives available to them.

***Distinguishing between the nineth, tenth, eleventh, and twelfth approaches:*** By eliminating obstacles to healthcare, we refer to the systematic efforts of healthcare providers to overcome barriers at multiple levels: individual, institutional, and societal. Given the multiplicity and variability of these barriers, healthcare providers must develop their own innovative solutions. Therefore, eliminating obstacles encompasses a wide range of activities rather than a single effort. Although other approaches also contribute to eliminating obstacles, they emerged as distinct themes because they represent specific, focused strategies that complement broader systemic efforts by providing immediate and individualized solutions.

For instance, leveraging personal connections is particularly significant for facilitating specialty care. The strategy of “pushing limits” involves healthcare providers stepping out of their regular routines. This approach emerged as a distinct theme because it presents unique ethical challenges that must be addressed to balance professional duties and personal lives. Providing care whenever possible requires healthcare providers to be resourceful and adaptable, strategically utilizing existing health policies to benefit undocumented migrants, even if those policies were not specifically legislated for this purpose.

***Distinguishing between the thirteenth and fourteenth approaches:*** Both approaches tackle financial barriers. However, the practice of healthcare providers offering care without any reimbursement or covering expenses themselves has emerged as a distinct theme due to its unique ethical challenges, which can result in financial strain on the providers. Consequently, we believe these two approaches differ in terms of impact, sustainability, and associated challenges. The thirteenth approach may have a broader impact, be more sustainable, and pose fewer challenges compared to the fourteenth approach.

## Discussion

This paper presents a scoping review investigating the advocacy approaches employed by healthcare providers in addressing the healthcare needs of undocumented migrants and advocating for their rights. Additionally, we pinpoint the ethical challenges encountered by these providers while fulfilling their advocacy roles. To facilitate discussion, it will be organized into two main parts: first, an examination of the advocacy approaches employed, followed by a brief reflection on healthcare providers' empowerment; and second, a reflection on the ethical challenges that have arisen.

### First: Advocacy approaches employed by healthcare providers

In the realm of healthcare provision for undocumented migrants, our findings emphasize the crucial role of "advocacy" as a transformative initiative. In certain scenarios, it transcends necessity, becoming an imperative action for healthcare providers. The pivotal role of advocacy arises from its potential to profoundly alter and enhance healthcare outcomes for undocumented migrants. By revisiting the adopted advocacy strategies and illustrative examples, we discern that the common outcome was ensuring healthcare accessibility for this patient cohort in various ways. Advocacy transcends mere necessity, since without it, the healthcare needs of undocumented migrants may not be adequately addressed, potentially leading to adverse health outcomes. The transformative effect of advocacy influences not only undocumented migrants but also healthcare providers, as the adoption of these practices enables them to perform their duties towards their patients more effectively. This pivotal role places healthcare providers in a unique position as the primary advocates for undocumented migrants.

Healthcare providers, positioned as the solitary champions for undocumented migrants and driven by the structured vulnerability imposed on those migrants, find themselves at the forefront of advocacy (Armin 2019). The attribution of vulnerability may be misunderstood when observed from an external perspective (Ogle 1997). Nevertheless, when healthcare providers employed the term “vulnerable”, they were unconsciously alluding to the “expressed vulnerability” conveyed by undocumented migrants themselves. In this context, vulnerability refers to the precarious and disadvantaged conditions that undocumented migrants face, which are often a result of systemic issues and structural inequities. This recognition of vulnerability stemmed from direct interactions between healthcare providers and migrants, as they endeavored to uncover the experiences and emotions of undocumented migrants (López-Domene et al. 2019; Granero-Molina et al. 2022; Vanobberghen et al. 2022). Empathetic interactions with undocumented migrants enable healthcare providers to gain a nuanced understanding of their vulnerability and subsequently advocate more effectively and appropriately for their health needs.

When healthcare providers observe the vulnerability of undocumented migrants from an external perspective, they might only see surface-level issues or stereotypical images of undocumented migrants without understanding the deeper, systemic factors contributing to their vulnerability. They might assume vulnerability is solely due to individual choices or circumstances rather than recognizing it as a result of broader social, economic, and political forces. This is why empathy is considered crucial, to comprehend the emotional and psychological toll that undocumented migrants experience due to living in constant fear of deportation, facing discrimination, or being denied basic rights and services. Without this empathetic understanding, the advocacy efforts of healthcare providers may fall short of addressing the specific health needs of undocumented migrants.

Delving into the included studies, the impact of advocacy emerges as a catalyst for healthcare providers to extend their services to a spectrum of marginalized groups of undocumented migrants. From cancer patients (Armin 2019), and expectant mothers (Fabi and Taylor 2019), to victims of human trafficking (López-Domene et al. 2019), and those grappling with mental health conditions (Mladovsky 2023), advocacy becomes the key to unlocking access to services that would otherwise remain out of reach.

This holds especially true for undocumented migrants, encapsulating the harsh reality they endure—a reality marked by challenging circumstances and often unjust limitations to healthcare access. Healthcare providers, intimately acquainted with the unjust challenges faced by this marginalized group, consciously elect to advocate for their health rights. In making this conscientious decision, healthcare providers stand as unwavering believers in rectifying the injustices afflicting undocumented migrants. Their advocacy is not merely a professional obligation but a manifestation of empathy, a response to the palpable unfairness that defines the lives of these individuals. In line with the findings of Laari et al., professional obligation, empathy for patients, and the vulnerability of patients were identified as factors facilitating nurses in their advocacy roles toward the less privileged and disadvantaged patients (Laari and Duma 2021). Additionally, interviewed nurses highlighted other motivating factors not covered in our scoping review, including societal norms and religious beliefs (Laari and Duma 2021).

After conducting our scoping review, it becomes apparent the diversity of advocacy initiatives. Some initiatives may entail voluntary participation in delivering healthcare to undocumented migrants. Others may be driven by the level of empathy and comprehension demonstrated by healthcare providers towards undocumented migrants, offering an avenue for personal development. Lastly, certain initiatives are concentrated on healthcare advocacy, aiming to creatively bridge barriers to healthcare for undocumented migrants. Despite the variety in adopted advocacy approaches, these strategies were largely consistent across different healthcare settings, such as governmental versus humanitarian, and across various professional backgrounds. This consistency may suggest a widespread nature of the identified advocacy approaches, regardless of the specific healthcare environments or professional roles of the providers.

Advocacy for the rights of undocumented immigrants goes beyond immediate medical care, addressing broader concerns related to their legal rights. This includes referring undocumented patients to legal services and connecting them with advocacy and informational organizations to help them understand and assert their rights, such as the right not to open the door to federal agents without a valid judicial warrant. While physicians should avoid endorsing specific private-practice attorneys, they can provide a list of reputable nonprofit organizations that offer pro bono or sliding-scale legal services (Kuczewski et al. 2019).

Given the intricate healthcare needs of undocumented migrants, healthcare providers must be cognizant of the challenges inherent in their advocacy roles and be aware of the associated risks, regardless of the chosen advocacy approach. This observation aligns with Cleaveland's findings, where social work advocacy efforts for undocumented Mexican migrants in the USA were deemed challenging due to their ineligibility for health insurance, food stamps, cash assistance, or federal housing. Nevertheless, despite these obstacles, opportunities to support these migrants were also identified (Cleaveland 2010).

Within the realm of health advocacy for undocumented migrants, despite the array of approaches employed, a common thread emerged—they all were triggered by a “response” to a “recognized issue”. In this sphere, the prominent challenge lies in the restricted or limited accessibility to healthcare services for undocumented migrants, a predicament widely acknowledged.

This issue comes to the forefront through direct interactions healthcare providers have with these migrants. Consequently, the impetus to engage in advocacy is intricately tied to the perceptions healthcare professionals hold regarding undocumented migrants. Therefore, the choice to engage in advocacy may be shaped by these perceptions. If healthcare providers view undocumented migrants as illegal, irrational, intruders, or as a strain on the healthcare system (Goldade 2009), it could negatively influence their inclination to advocate for the health rights of undocumented migrants. This aligns with the concept of "othering theory" that examines how marginalized groups such as undocumented migrants are portrayed as fundamentally different from the dominant group, leading to their exclusion and disconnection from host communities (Grove and Zwi 2006). Therefore, healthcare providers' perceptions of undocumented migrants as "the other" may hinder their advocacy efforts.

In addition to healthcare providers' perceptions of undocumented migrants, numerous obstacles may impact their willingness to advocate the health rights of this patient group. Our scoping review identified some of these barriers, such as healthcare providers citing family commitments and time constraints (Granero-Molina et al. 2021). Societal biases or attitudes towards undocumented migrants were also underscored (Marrow 2012; Bianchi et al. 2019; Tiedje and Plevak 2014), potentially exerting a negative influence on healthcare providers' advocacy decisions. Furthermore, the absence of legal entitlement for undocumented migrants could cast doubt on the legitimacy of healthcare advocacy efforts, as medical aid might be subject to criminalization (Castañeda 2011). Beyond these mentioned hurdles, other factors may contribute, including the interference of immigration officers in advocacy endeavors (Yu et al. 2020), and patient-related challenges, such as undocumented migrants lacking trust in healthcare providers (Teunissen et al. 2015; Gullberg and Wihlborg 2014).

### Reflection on healthcare providers' empowerment

In this paper, we have briefly mentioned the term “empowering healthcare providers” on several occasions. Here, we reflect on our understanding of this empowerment within the context of health advocacy and explain its connection to the advocacy approaches identified, as well as its impact on healthcare delivery for undocumented migrants.

Advocacy initiatives generally pursue two main objectives: safeguarding vulnerable populations through representational advocacy and empowering disadvantaged groups through facilitational advocacy (Carlisle 2000). In the context of health advocacy for undocumented migrants, we find that empowering healthcare providers is equally important to empowering the undocumented migrants themselves.

While numerous models of healthcare provider empowerment exist, we focus here on the concept of “enabling” providers to perform their roles effectively within the constraints imposed on them. This includes enabling them to provide equal access to healthcare services, regardless of a patient’s immigration or insurance status. When discussing empowerment, there is often greater emphasis on structural empowerment, which refers to external factors and conditions that facilitate empowerment. These factors include organizational structures that provide healthcare providers with adequate resources, leadership support, and opportunities for professional growth and development (De Carvalho et al. 2017; Wagner et al. 2010).

However, our scoping review revealed that healthcare providers often derived a sense of empowerment internally. In some cases, this empowerment stemmed from their compassion for undocumented migrants, which motivated them to take action. In other cases, it emerged out of necessity, due to a lack of structural empowerment. Consequently, providers often developed their own approaches to fulfill their roles in caring and advocating for undocumented migrant patients. This paper highlighted several examples of how these self-initiated approaches contributed to enhancing healthcare accessibility for undocumented migrants.

We find that the advocacy approaches identified in this scoping review are directly linked to healthcare provider empowerment. These approaches empower providers to challenge and mitigate barriers to healthcare provision for undocumented migrants. This connection is particularly evident in approaches such as voluntary care, understanding and empathy, and systemic health advocacy. For example, voluntary care empowers providers to address immediate healthcare needs by offering services outside of institutional constraints. Similarly, understanding and empathy empower healthcare providers to offer culturally sensitive, holistic care that enhances the overall quality of care delivered. Systemic health advocacy empowers healthcare providers to navigate restrictive systems and overcome economic obstacles to care provision. Beyond these means, we find these advocacy approaches also contribute to the psychological empowerment of healthcare providers. Psychological empowerment refers to an internal process in which providers experience feelings of autonomy, meaning, and impact in their professional roles (Wagner et al. 2010).

Although the connection between empowerment and advocacy approaches grouped under personal growth and awareness may seem less direct, we find that these approaches have a synergistic effect when combined with other forms of advocacy. Personal growth contributes to empowerment by helping healthcare providers develop a deeper sense of purpose, thereby increasing their likelihood of engaging in sustained advocacy efforts, such as participation in voluntary networks and migrant clinics (Holmes 2012; Sandblom and Mangrio 2017). These challenging roles demand significant commitment and inspiration, and providers who feel inspired are more likely to experience a sense of self-efficacy and motivation—key components of effective healthcare advocacy. Self-efficacy refers to the confidence healthcare providers have in their ability to competently perform tasks. Providers with higher self-efficacy are more likely to adapt to challenging environments and engage in effective communication, empathetic interactions, and patient-centered care (Huang et al. 2022).

Personal growth further empowers healthcare providers by honing their skills and broadening their perspectives. For instance, several providers noted that working with migrant populations helped them overcome pre-existing stereotypes, thereby reinforcing their commitment to advocacy roles (Jiménez-Lasserrotte et al. 2023). This growth not only strengthens providers’ sense of purpose but also underscores the importance of developing key competencies, such as communication and trust-building, which are essential for meaningful empowerment. For empowerment to be truly effective, healthcare providers need to be competent not just in medical knowledge but also in interpersonal skills. Empowering providers with these competencies enables them to engage more effectively in empowering patients, contributing to their own professional empowerment as they view themselves as partners in the health journey, rather than as authoritative figures (Halvorsen et al. 2020).

### Second: Reflection on the ethical challenges that emerged

According to our analysis, sixteen ethical challenges have been identified. We will briefly reflect on these challenges.

Numerous healthcare providers have expressed a sincere willingness to volunteer and address the healthcare needs of undocumented migrants. However, a potential conflict may arise between their eagerness to help and the imperative for professional competence (Castañeda 2011). Healthcare providers engaged in advocacy efforts need to recognize that acting as advocates, while fulfilling and meaningful, can present ethical challenges if unreflective advocacy results in the unfair or poorly planned allocation of resources on an ad hoc basis (Berlinger and Raghavan 2013). Additionally, they must weigh ethical principles to safeguard both the well-being of the migrants and the professional integrity of the advocates themselves. This involves demonstrating respect for the autonomy of undocumented migrants by ensuring they are fully informed about the nature of the care they will receive, including any potential risks or unconventional circumstances.

In challenging environments or non-traditional settings, healthcare providers face a heightened responsibility to uphold the principle of informed consent, encompassing transparent communication about the limitations of care and the voluntary nature of the healthcare provider's involvement. Moreover, in addition to respecting the autonomy of undocumented migrants, considerations of beneficence and non-maleficence are crucial, involving efforts to maximize benefits, eliminate injustice and deliver the highest quality care within the given constraints (Kluesner et al. 2021).

One advocacy approach embraced by healthcare providers involved developing a heightened understanding of the emotions experienced by undocumented migrants. However, within this approach, ethical considerations also surfaced, particularly focusing on the significance of respecting the privacy of undocumented migrants and cultivating trust in the process (Kvamme and Voldner 2022; Tiedje and Plevak 2014; Doshi et al. 2020). The legal status of undocumented migrants often leaves them in a constant state of fear, making it imperative to prioritize the preservation of their privacy to establish a secure and trusting environment (Hoekstra 2021).

The importance of privacy extends beyond the physical space, encompassing personal information, experiences, and emotions. When healthcare is delivered to undocumented migrants, they navigate unfamiliar territory, providing care in conditions they have not encountered before (Vanobberghen et al. 2022). In such instances, the significance of respecting privacy and fostering trust becomes even more crucial, considering the heightened vulnerability of migrants, both medically and emotionally (Sandblom and Mangrio 2017). To build trust and communicate effectively with undocumented migrants, healthcare providers must grasp the concepts of recognition and respect. This involves acknowledging the dignity and humanity of undocumented migrants, thereby creating an environment where they feel safe and valued. This encourages them to express their needs and concerns (Andersson and Punzi 2024).

Healthcare providers must be attuned to the possibility that migrants may have valid reasons for concealing certain aspects of their lives, and this need for privacy should be acknowledged and respected (Kvamme and Voldner 2022). Additionally, healthcare providers should be mindful of the inherent power dynamics in the caregiver-patient relationship, striving to create an environment that is non-coercive and supportive. Neglecting to respect privacy can lead to an erosion of trust and compromise the integrity of the healthcare provider-patient relationship (Sahraoui 2020).

Another ethical challenge identified is the potential conflict between healthcare providers' individual values and their institutional responsibilities (Lafaut 2021). Healthcare professionals often join the field with a strong dedication to delivering fair care to all patients, particularly marginalized individuals, and may feel a personal duty to advocate for the rights of underserved populations (Stephen and Zoucha 2020). This commitment may extend beyond the confines of institutional policies. Within this framework, Kuczewski outlined two generations of ethical challenges. The first generation revolves around finding ethical ways to meeting the undocumented migrants' health needs within limited resources and ethically discharge them after healthcare provision. Meanwhile, the second generation focuses on facilitating accessibility to available health resources for undocumented migrants. Kuczewski argues that organizational and professional values such as care, efficiency, and the promotion of public health apply to both generations of issues (Kuczewski 2019). Rodriguez also adds the necessity of considering a moral or ethical theory rooted in human rights (Rodriguez 2019).

While operating within institutional frameworks that dictate policies, regulations, and limitations, healthcare providers must contend with defined roles and responsibilities. These institutions, whether governmental or non-governmental, set parameters for healthcare providers (Sahraoui 2020; Cervantes et al. 2018). The conflict arises when personal values clash with institutional roles, creating tension between the desire to offer comprehensive care and the constraints imposed by organizational dynamics (Cervantes et al. 2018). To address this clash, healthcare providers must navigate it by devising inventive, problem-solving strategies. This involves finding creative solutions to deliver appropriate care within the limitations imposed by their roles and the dynamics of their institutions (Fabi and Taylor 2019; Mladovsky 2023). Successfully navigating these limitations necessitates a thorough understanding of both the personal ethical commitments of the healthcare providers and the institutional constraints within which they operate. While healthcare providers may develop their own methods to maneuver within the system and accomplish their tasks, the relevant motives may encompass efficiency, problem-solving, fairness, and more adversely avoidance or relocation of problems (Berlinger 2019).

At the institutional level, healthcare providers can create safe and migrant-friendly environments by minimizing cooperation with immigration enforcement to protect patients' rights. This includes designating private areas within healthcare facilities to ensure patient privacy and limit unauthorized law enforcement access, maintaining the confidentiality of patient information, especially regarding immigration status, informing patients of their privacy rights, and only collecting and recording immigration status when absolutely necessary (Saadi 2020). While knowing a patient's immigration status can enhance the quality of care, explicitly documenting this information can lead to discrimination and legal complications. To mitigate these risks, healthcare providers can use indirect language, such as “immigration stressors”, and emphasize verbal communication with other providers. These strategies help foster a safe and non-discriminatory healthcare environment (Kim et al. 2019).

Last but not least, based on our scoping review, healthcare providers may stretch their limits to provide care for undocumented migrants. The adoption of this strategy may present healthcare providers with difficulties in establishing and preserving distinct personal boundaries (Willen 2011; Doshi et al. 2020; Hoekstra 2021). They might struggle to balance their commitment to advocacy with the imperative for self-care, potentially leading to burnout (Cervantes et al. 2018; Armin 2019). Overextending oneself without clear personal boundaries can lead to emotional exhaustion and compassion fatigue (Armin 2019; Vanobberghen et al. 2022). Healthcare providers should be mindful of their capacity limits and ensure that their dedication to advocacy does not jeopardize their mental, emotional, and physical well-being.

To establish and uphold clear personal boundaries, healthcare providers might consider the following: engaging in regular self-reflection to comprehend their limits, values, and emotional reactions to advocacy efforts; being conscious of personal vulnerabilities and stressors to identify areas where boundaries may require reinforcement; delineating the scope of their advocacy work and setting specific limits on how far they can push their professional and personal boundaries; prioritizing self-care practices; setting realistic goals for advocacy efforts by considering the needs of the undocumented migrant population and the provider's capacity; seeking support; and developing coping strategies. Furthermore, healthcare providers should acknowledge that maintaining personal well-being is not only crucial for the advocate but also contributes to the sustainability and effectiveness of advocacy efforts.

### Contributions and implications

The primary contribution of this study lies in its systematic identification and categorization of advocacy approaches and ethical challenges by presenting concrete examples of how healthcare providers navigate the complexities of delivering care to undocumented migrants. Additional contributions of this study might include:

- Offering a structured framework for understanding and implementing advocacy in healthcare delivery for undocumented migrants, serving as a guide for healthcare providers who may be uncertain about integrating advocacy into their daily routines.
- Providing practical examples that could inform training programs aimed at improving healthcare delivery to undocumented migrants.
- Highlighting the need for structured support and clear guidelines to help providers navigate the ethical challenges of their advocacy efforts, ensuring these efforts are both effective and ethically sound.

Our findings not only highlight the proactive roles healthcare providers can adopt but also emphasize:

- The need for comprehensive training programs that equip healthcare providers with both the skills and ethical frameworks required for effective advocacy.
- The importance of institutional support in fostering an environment where healthcare providers feel empowered, and advocacy can thrive without compromising professional responsibilities.
- The need for healthcare systems to adopt more holistic and inclusive approaches, incorporating advocacy as a core component of healthcare provision to undocumented migrants.

## Limitations and strengths

The criteria for inclusion were specifically confined to qualitative papers published exclusively in English. This limitation may lead to a narrowed portrayal of advocacy efforts across diverse cultural contexts, potentially introducing a cultural bias and thereby impacting the generalizability of the findings. Similarly, we did not enforce any restrictions on the geographic region of publication. Nevertheless, the majority of the incorporated studies were conducted mainly on two continents: North America and Europe, specifically within the United Nations’ regional group of Western European and other States. While this does not necessarily constitute a direct limitation of our inclusion and exclusion criteria, it does result in regional variations. It is important to note that undocumented migrant populations encounter distinct challenges in different regions, and healthcare advocacy strategies may be influenced by national policies and laws, and socioeconomic factors. Furthermore, the demographics of undocumented migrants, including their country of origin, as well as age and gender distribution can differ among various countries. Therefore, tailored advocacy strategies may be necessary to address the distinct health needs of diverse subgroups within the undocumented migrant population.

In our scoping review, we deliberately refrained from imposing restrictions on publication dates. While some may argue that older studies could be influenced by distinct contextual factors and evolving healthcare landscapes, the relevance of such considerations largely hinges on the specific objectives of the review undertaken. In our investigation, we contend that this approach did not introduce publication bias, as our primary goal was to uncover advocacy approaches adopted by healthcare providers in addressing the constrained access of undocumented migrants to healthcare services. Given the globally acknowledged persistence of the issue of restricted healthcare availability, we deemed it crucial to include older publications in our analysis. This inclusion aimed to discern any potential variations in advocacy strategies over time. Notably, our scrutiny, particularly when examining the USA as a case study, revealed a consistency in advocacy approaches across different periods, as evidenced by eleven studies conducted in the country. Consequently, we assert that the incorporation of older publications was a strength rather than a limitation. This is underscored by the enduring relevance of advocacy efforts, which, in our perspective, have no expiration date given the ongoing nature of the issue. Consequently, these historical approaches provide valuable practical guidance for contemporary healthcare providers grappling with similar challenges in ensuring healthcare access for undocumented migrants.

In our analysis, we identified diverse advocacy approaches and intercorrelated ethical challenges. While the ethical challenges were less saturated in the included papers compared to the advocacy approaches, this should not diminish the importance of the identified advocacy approaches. These approaches not only offer practical guidance for healthcare providers to effectively champion the health rights of undocumented migrant patients but also contribute to raising awareness of the ethical challenges that may arise in their advocacy roles.

## Conclusion

This scoping review examined qualitative evidence to uncover the advocacy approaches employed by healthcare providers in their efforts to improve access to healthcare services for undocumented migrants. Furthermore, we pinpointed the ethical challenges associated with these approaches within this specific context. Our findings indicate that healthcare providers consciously chose to engage in advocacy, driven by an internal sense of empowerment that extended beyond their professional duties, in response to the tangible injustices faced by undocumented migrants. The spectrum of advocacy initiatives varied, encompassing voluntary participation, empathetic understanding, and healthcare-focused strategies, all stemming from a response to the acknowledged issue of limited accessibility to healthcare services.

Our scoping review also brought to the forefront several ethical challenges demanding that healthcare providers strike a delicate balance between their eagerness to assist and their professional competence, while simultaneously respecting the autonomy of undocumented migrants and navigating potential conflicts between individual values and institutional responsibilities. Furthermore, the significance of privacy, trust, and acknowledgment of power dynamics in the caregiver-patient relationship was underscored, with a recognition of the heightened vulnerability of undocumented migrants. The review also delved into the risk of healthcare providers overextending themselves, underscoring the importance of establishing and preserving personal boundaries for the well-being of advocates and the sustainability of advocacy endeavors.

## Supplementary Information

Below is the link to the electronic supplementary material.

Supplementary file1 (DOCX 49 KB)

Supplementary file2 (DOCX 18 KB)

Supplementary file3 (DOCX 17 KB)

Supplementary file4 (DOCX 37 KB)

## Data Availability

All essential data are available in supplementary materials.

## Footnotes

1 In this scoping review, we emphasize advocacy initiatives at the level of “cases”, particularly those adopted by healthcare providers to promote the health of undocumented migrants. Additionally, we focus on “agency” activities that involve working the system to address health disparities and eliminate obstacles to achieve equal access to healthcare for this cohort of patients.

2 By empowering healthcare providers, we refer to enhancing their capacity to fulfill their roles, including their advocacy roles, to better address the health needs of undocumented migrants and improve their access to healthcare services, even in challenging healthcare environments and despite organizational and systemic obstacles.

3 The PICO Framework is traditionally used in quantitative research to frame specific and measurable components, while qualitative studies require a more flexible framework to capture their exploratory nature. In our scoping review, the complexity and interconnectedness of advocacy strategies and ethical challenges can blur the distinctions typically emphasized in the PICO framework. Despite this potential obstacle, the structured approach of the PICO framework proved highly beneficial at various stages of our study, from question formulation to data analysis and reporting. By adopting this framework, we were able to: (1) make our research question more focused and manageable; (2) define the key elements of our study (population, intervention, and outcomes) and develop a structured search strategy; (3) maintain a clear focus throughout the research process, aiding readers in understanding the scope and objectives of our review; (4) organize the synthesis of qualitative evidence by systematically categorizing and comparing findings across different studies; and (5) enhance the rigor and transparency of our scoping review, thereby improving its credibility and replicability by ensuring that all elements of our study were systematically considered and reported.

4 The term "third-world" is a direct quotation. Otherwise, we prefer using other terms to avoid cultural bias.
