# Supplementary material for: Healthcare providers' advocacy approaches and ethical challenges in delivering healthcare to undocumented migrants: a scoping review
Source: Med Health Care Philos. 2024 Oct 7;27(4):579–606. doi: 10.1007/s11019-024-10225-8 (PMC11519158; doi:10.1007/s11019-024-10225-8)
Supplement: Supplementary file 1 — Supplementary file1 (DOCX 49 KB) [file 11019_2024_10225_MOESM1_ESM.docx]

***Appendix One:*** Search strategy for the scoping review by using four databases (Medline, Embase, Cinahl, and Cochrane Library)

***Table One:*** Search strategy by using Medline/Ovid and the total number of retrieved records for each search query categorized according to the main search blocks (population, intervention, and outcome). Date of search: 12-Mar-23

| **No.** | **Search query** | **Total no. of retrieved records** |
| --- | --- | --- |
| ***First search block: Population (undocumented migrants)*** | | |
| 1 | exp undocumented immigrants/ | 521 |
| 2 | undocumented.ab,kw,ti. | 4297 |
| 3 | unauthorized immigrant$.ab,kw,ti. | 70 |
| 4 | unauthorized migrant$.ab,kw,ti. | 28 |
| 5 | unauthorized worker$.ab,kw,ti. | 4 |
| 6 | illegal immigrant$.ab,kw,ti. | 226 |
| 7 | illegal migrant$.ab,kw,ti. | 325 |
| 8 | illegal worker$.ab,kw,ti. | 20 |
| 9 | irregular migrant$.ab,kw,ti. | 85 |
| 10 | irregular immigrant$.ab,kw,ti. | 21 |
| 11 | irregular worker$.ab,kw,ti. | 5 |
| 12 | rejected asylum seeker$.ab,kw,ti. | 9 |
| 13 | 1 or 2 or 3 or 4 or 5 or 6 or 7 or 8 or 9 or 10 or 11 or 12  exp undocumented immigrants/ or undocumented.ab,kw,ti. or unauthorized immigrant$.ab,kw,ti. or unauthorized migrant$.ab,kw,ti. or unauthorized worker$.ab,kw,ti. or illegal immigrant$.ab,kw,ti. or illegal migrant$.ab,kw,ti. or illegal worker$.ab,kw,ti. or irregular migrant$.ab,kw,ti. or irregular immigrant$.ab,kw,ti. or irregular worker$.ab,kw,ti. or rejected asylum seeker$.ab,kw,ti. | 5053 |
| ***Second search block: Intervention (Patient advocacy strategies employed by healthcare providers in the process of delivering healthcare services)*** | | |
| 14 | exp "delivery of health care"/ | 1219341 |
| 15 | exp health services accessibility/ | 133764 |
| 16 | exp healthcare disparities/ | 21832 |
| 17 | exp right to health/ | 239 |
| 18 | exp patient advocacy/ | 24201 |
| 19 | exp physician-patient relations/ | 76029 |
| 20 | exp health personnel/ | 604205 |
| 21 | exp physicians/ | 174022 |
| 22 | exp allied health personnel/ | 53820 |
| 23 | personnel.ab,kw,ti. | 85817 |
| 24 | physician$.ab,kw,ti. | 451642 |
| 25 | doctor$.ab,kw,ti. | 149121 |
| 26 | nurse$.ab,kw,ti. | 317280 |
| 27 | health provider$.ab,kw,ti. | 8778 |

***Table One:*** Continued.

| 28 | health worker$.ab,kw,ti. | 23480 |
| --- | --- | --- |
| 29 | professionals.ab,kw,ti. | 183501 |
| 30 | healthcare.ab,kw,ti. | 316541 |
| 31 | care.ab,kw,ti. | 1726673 |
| 32 | therapy.ab,kw,ti. | 2102412 |
| 33 | treatment.ab,kw,ti. | 5100110 |
| 34 | 14 or 15 or 16 or 17 or 18 or 19 or 20 or 21 or 22 or 23 or 24 or 25 or 26 or 27 or 28 or 29 or 30 or 31 or 32 or 33  exp "delivery of health care"/ or exp health services accessibility/ or exp healthcare disparities/ or exp right to health/ or exp patient advocacy/ or exp physician-patient relations/ or exp health personnel/ or exp physicians/ or exp allied health personnel/ or personnel.ab,kw,ti. or physician$.ab,kw,ti. or doctor$.ab,kw,ti. or nurse$.ab,kw,ti. or health provider$.ab,kw,ti. or health worker$.ab,kw,ti. or professionals.ab,kw,ti. or healthcare.ab,kw,ti. or care.ab,kw,ti. or therapy.ab,kw,ti. or treatment.ab,kw,ti. | 8802949 |
| ***Third search block: Outcome (ethical challenges)*** | | |
| 35 | exp ethics/ | 155152 |
| 36 | exp bioethics/ | 11740 |
| 37 | exp moral obligations/ | 6837 |
| 38 | exp ethics, clinical/ | 64690 |
| 39 | exp ethics, institutional/ | 3027 |
| 40 | exp jurisprudence/ | 215854 |
| 41 | exp ethics committees, clinical/ | 2336 |
| 42 | exp "codes of ethics"/ | 5499 |
| 43 | exp ethics consultation/ | 1375 |
| 44 | exp principle-based ethics/ | 32727 |
| 45 | exp ethics committees/ | 9901 |
| 46 | exp ethics, professional/ | 74615 |
| 47 | exp ethics, nursing/ | 10521 |
| 48 | exp ethics, medical/ | 48399 |
| 49 | exp ethical theory/ | 3604 |
| 50 | ethic$.ab,kw,ti. | 160238 |
| 51 | bioethic$.ab,kw,ti. | 17830 |
| 52 | moral$.ab,kw,ti. | 40422 |
| 53 | "professional obligation$".ab,kw,ti. | 600 |
| 54 | responsabilit$.ab,kw,ti. | 450 |
| 55 | issue$.ab,kw,ti. | 680831 |
| 56 | challenge$.ab,kw,ti. | 876354 |
| 57 | dilemma$.ab,kw,ti. | 44458 |

***Table One:*** Continued.

| 58 | 35 or 36 or 37 or 38 or 39 or 40 or 41 or 42 or 43 or 44 or 45 or 46 or 47 or 48 or 49 or 50 or 51 or 52 or 53 or 54 or 55 or 56 or 57  exp ethics/ or exp bioethics/ or exp moral obligations/ or exp ethics, clinical/ or exp ethics, institutional/ or exp jurisprudence/ or exp ethics committees, clinical/ or exp "codes of ethics"/ or exp ethics consultation/ or exp principle-based ethics/ or exp ethics committees/ or exp ethics, professional/ or exp ethics, nursing/ or exp ethics, medical/ or exp ethical theory/ or ethic$.ab,kw,ti. or bioethic$.ab,kw,ti. or moral$.ab,kw,ti. or "professional obligation$".ab,kw,ti. or responsabilit$.ab,kw,ti. or issue$.ab,kw,ti. or challenge$.ab,kw,ti. or dilemma$.ab,kw,ti. | 1901608 |
| --- | --- | --- |
| ***Final search query: Combining the three search blocks by Boolean operator “and”*** | | |
| 59 | 13 and 34 and 58  (exp undocumented immigrants/ or undocumented.ab,kw,ti. or unauthorized immigrant$.ab,kw,ti. or unauthorized migrant$.ab,kw,ti. or unauthorized worker$.ab,kw,ti. or illegal immigrant$.ab,kw,ti. or illegal migrant$.ab,kw,ti. or illegal worker$.ab,kw,ti. or irregular migrant$.ab,kw,ti. or irregular immigrant$.ab,kw,ti. or irregular worker$.ab,kw,ti. or rejected asylum seeker$.ab,kw,ti.) and (exp "delivery of health care"/ or exp health services accessibility/ or exp healthcare disparities/ or exp right to health/ or exp patient advocacy/ or exp physician-patient relations/ or exp health personnel/ or exp physicians/ or exp allied health personnel/ or personnel.ab,kw,ti. or physician$.ab,kw,ti. or doctor$.ab,kw,ti. or nurse$.ab,kw,ti. or health provider$.ab,kw,ti. or health worker$.ab,kw,ti. or professionals.ab,kw,ti. or healthcare.ab,kw,ti. or care.ab,kw,ti. or therapy.ab,kw,ti. or treatment.ab,kw,ti.) and (exp ethics/ or exp bioethics/ or exp moral obligations/ or exp ethics, clinical/ or exp ethics, institutional/ or exp jurisprudence/ or exp ethics committees, clinical/ or exp "codes of ethics"/ or exp ethics consultation/ or exp principle-based ethics/ or exp ethics committees/ or exp ethics, professional/ or exp ethics, nursing/ or exp ethics, medical/ or exp ethical theory/ or ethic$.ab,kw,ti. or bioethic$.ab,kw,ti. or moral$.ab,kw,ti. or "professional obligation$".ab,kw,ti. or responsabilit$.ab,kw,ti. or issue$.ab,kw,ti. or challenge$.ab,kw,ti. or dilemma$.ab,kw,ti.) | 603 |
| 60 | limit 59 to English language | 571 |
| The selected source is Ovid MEDLINE(R) and Epub Ahead of Print, In-Process, In-Data-Review & Other Non-Indexed Citations and Daily 1946 to March 10, 2023  Note: / = subject heading, exp = exploded subject heading, ab = abstract, kw = keyword heading, and ti = title  The dollar sign ($) is used for truncation  Double quotation marks (“ ”) around a phrase refer that the words should be present in the same sequence as in the search phrase  The Boolean operator (or) is used to expand a search by broadening the set and indicates that each result contains at least one search term  The Boolean operator (and) is used to narrow a search by linking different concepts or search blocks and indicates that each result contains all search terms or queries | | |

***Table Two:*** Search strategy by using Embase and the total number of retrieved records for each search query categorized according to the main search blocks (population, intervention, and outcome). Date of search: 12-Mar-23

| **No.** | **Search query** | **Total no. of retrieved records** |
| --- | --- | --- |
| ***First search block: Population (undocumented migrants)*** | | |
| 1 | 'undocumented immigrant'/exp | 832 |
| 2 | undocumented:ab,ti | 5322 |
| 3 | 'unauthorized immigrant*':ab,ti | 57 |
| 4 | 'unauthorized migrant*':ab,ti | 22 |
| 5 | 'unauthorized worker*':ab,ti | 3 |
| 6 | 'illegal immigrant*':ab,ti | 230 |
| 7 | 'illegal migrant*':ab,ti | 64 |
| 8 | 'illegal worker*':ab,ti | 16 |
| 9 | 'irregular migrant*':ab,ti | 74 |
| 10 | 'irregular immigrant*':ab,ti | 27 |
| 11 | 'irregular worker*':ab,ti | 8 |
| 12 | 'rejected asylum seeker*':ab,ti | 5 |
| 13 | 1 or 2 or 3 or 4 or 5 or 6 or 7 or 8 or 9 or 10 or 11 or 12  'undocumented immigrant'/exp OR undocumented:ab,ti OR 'unauthorized immigrant*':ab,ti OR 'unauthorized migrant*':ab,ti OR 'unauthorized worker*':ab,ti OR 'illegal immigrant*':ab,ti OR 'illegal migrant*':ab,ti OR 'illegal worker*':ab,ti OR 'irregular migrant*':ab,ti OR 'irregular immigrant*':ab,ti OR 'irregular worker*':ab,ti OR 'rejected asylum seeker*':ab,ti | 6031 |
| ***Second search block: Intervention (Patient advocacy strategies employed by healthcare providers in the process of delivering healthcare services)*** | | |
| 14 | 'health care delivery'/exp | 4154806 |
| 15 | 'health care disparity'/exp | 21431 |
| 16 | 'patient advocacy'/exp | 23943 |
| 17 | 'professional-patient relationship'/exp | 171841 |
| 18 | 'health care personnel'/exp | 1933645 |
| 19 | personnel:ab,ti | 111780 |
| 20 | 'physician*':ab,ti | 649412 |
| 21 | 'doctor*':ab,ti | 216969 |
| 22 | 'nurse*':ab,ti | 389727 |
| 23 | 'health provider*':ab,ti | 11385 |
| 24 | 'health worker*':ab,ti | 27722 |
| 25 | professionals:ab,ti | 247234 |
| 26 | healthcare:ab,ti | 518072 |
| 27 | care:ab,ti | 2416018 |
| 28 | therapy:ab,ti | 3161837 |
| 29 | treatment:ab,ti | 7181077 |

***Table Two:*** Continued.

| 30 | 14 or 15 or 16 or 17 or 18 or 19 or 20 or 21 or 22 or 23 or 24 or 25 or 26 or 27 or 28 or 29  'health care delivery'/exp OR 'health care disparity'/exp OR 'patient advocacy'/exp OR 'professional-patient relationship'/exp OR 'health care personnel'/exp OR personnel:ab,ti OR 'physician*':ab,ti OR 'doctor*':ab,ti OR 'nurse*':ab,ti OR 'health provider*':ab,ti OR 'health worker*':ab,ti OR professionals:ab,ti OR healthcare:ab,ti OR care:ab,ti OR therapy:ab,ti OR treatment:ab,ti | 13707915 |
| --- | --- | --- |
| ***Third search block: Outcome (ethical challenges)*** | | |
| 31 | 'ethics'/exp | 349090 |
| 32 | 'professional standard'/exp | 564398 |
| 33 | 'morality'/exp | 43802 |
| 34 | 'jurisprudence'/exp | 35104 |
| 35 | ethic*:ab,ti | 229546 |
| 36 | bioethic*:ab,ti | 12073 |
| 37 | moral*:ab,ti | 45033 |
| 38 | 'professional obligation*':ab,ti | 675 |
| 39 | responsabilit*:ab,ti | 562 |
| 40 | issue*:ab,ti | 875013 |
| 41 | challenge*:ab,ti | 1077769 |
| 42 | dilemma*:ab,ti | 56407 |
| 43 | 31 or 32 or 33 or 34 or 35 or 36 or 37 or 38 or 39 or 40 or 41 or 42  'ethics'/exp OR 'professional standard'/exp OR 'morality'/exp OR 'jurisprudence'/exp OR ethic*:ab,ti OR bioethic*:ab,ti OR moral*:ab,ti OR 'professional obligation*':ab,ti OR responsabilit*:ab,ti OR issue*:ab,ti OR challenge*:ab,ti OR dilemma*:ab,ti | 2774348 |
| ***Final search query: Combining the three search blocks by Boolean operator “and”*** | | |
| 44 | 13 and 30 and 43  ('undocumented immigrant'/exp OR undocumented:ab,ti OR 'unauthorized immigrant*':ab,ti OR 'unauthorized migrant*':ab,ti OR 'unauthorized worker*':ab,ti OR 'illegal immigrant*':ab,ti OR 'illegal migrant*':ab,ti OR 'illegal worker*':ab,ti OR 'irregular migrant*':ab,ti OR 'irregular immigrant*':ab,ti OR 'irregular worker*':ab,ti OR 'rejected asylum seeker*':ab,ti) AND ('health care delivery'/exp OR 'health care disparity'/exp OR 'patient advocacy'/exp OR 'professional-patient relationship'/exp OR 'health care personnel'/exp OR personnel:ab,ti OR 'physician*':ab,ti OR 'doctor*':ab,ti OR 'nurse*':ab,ti OR 'health provider*':ab,ti OR 'health worker*':ab,ti OR professionals:ab,ti OR healthcare:ab,ti OR care:ab,ti OR therapy:ab,ti OR treatment:ab,ti) AND ('ethics'/exp OR 'professional standard'/exp OR 'morality'/exp OR 'jurisprudence'/exp OR ethic*:ab,ti OR bioethic*:ab,ti OR moral*:ab,ti OR 'professional obligation*':ab,ti OR responsabilit*:ab,ti OR issue*:ab,ti OR challenge*:ab,ti OR dilemma*:ab,ti) | 894 |
| 45 | 13 and 30 and 43 and [english]/lim | 868 |
| Note: / = search term in Emtree (the deep indexing of Embase), exp = exploded search term, ab = abstract, and ti = title  The asterisk (*) is used for truncation  Single quotation marks (‘ ’) around a phrase indicate that the words should be present in the same sequence as in the search phrase  The Boolean operator (or) is used to expand a search by broadening the set and indicates that each result contains at least one search term  The Boolean operator (and) is used to narrow a search by linking different concepts or search blocks and indicates that each result contains all search terms or queries | | |

***Table Three:*** Search strategy by using Cinahl via EBSCO host and the total number of retrieved records for each search query categorized according to the main search blocks (population, intervention, and outcome). Date of search: 12-Mar-23

| **No.** | **Search query** | **Total no. of retrieved records** |
| --- | --- | --- |
| ***First search block: Population (undocumented migrants)*** | | |
| 1 | (MH "Undocumented Immigrants") | 1085 |
| 2 | TI "undocumented" OR AB "undocumented" | 1753 |
| 3 | TI "unauthorized immigrant*" OR AB "unauthorized immigrant*" | 56 |
| 4 | TI "unauthorized migrant*" OR AB "unauthorized migrant*" | 15 |
| 5 | TI "unauthorized worker*" OR AB "unauthorized worker*" | 1 |
| 6 | TI "illegal immigrant*" OR AB "illegal immigrant*" | 84 |
| 7 | TI "illegal migrant*" OR AB "illegal migrant*" | 28 |
| 8 | TI "illegal worker*" OR AB "illegal worker*" | 5 |
| 9 | TI "irregular migrant*" OR AB "irregular migrant*" | 47 |
| 10 | TI "irregular immigrant*" OR AB "irregular immigrant*" | 9 |
| 11 | TI "irregular worker*" OR AB "irregular worker*" | 6 |
| 12 | TI "rejected asylum seeker*" OR AB "rejected asylum seeker*" | 2 |
| 13 | 1 or 2 or 3 or 4 or 5 or 6 or 7 or 8 or 9 or 10 or 11 or 12  (MH "Undocumented Immigrants") OR TI "undocumented" OR AB "undocumented" OR TI "unauthorized immigrant*" OR AB "unauthorized immigrant*" OR TI "unauthorized migrant*" OR AB "unauthorized migrant*" OR TI "unauthorized worker*" OR AB "unauthorized worker*" OR TI "illegal immigrant*" OR AB "illegal immigrant*" OR TI "illegal migrant*" OR AB "illegal migrant*" OR TI "illegal worker*" OR AB "illegal worker*" OR TI "irregular migrant*" OR AB "irregular migrant*" OR TI "irregular immigrant*" OR AB "irregular immigrant*" OR TI "irregular worker*" OR AB "irregular worker*" OR TI "rejected asylum seeker*" OR AB "rejected asylum seeker*" | 2522 |
| ***Second search block: Intervention (Patient advocacy strategies employed by healthcare providers in the process of delivering healthcare services)*** | | |
| 14 | (MH "Health Care Delivery+") | 402488 |
| 15 | (MH "Right to Health") | 200 |
| 16 | (MH "Patient Advocacy") | 14864 |
| 17 | (MH "Physician-Patient Relations") | 35677 |
| 18 | (MH "Health Personnel+") | 625580 |
| 19 | TI "personnel" OR AB "personnel" | 32655 |
| 20 | TI "physician*" OR AB "physician*" | 170496 |
| 21 | TI "doctor*" OR AB "doctor*" | 71537 |
| 22 | TI "nurse*" OR AB "nurse*" | 386409 |
| 23 | TI "health provider*" OR AB "health provider*" | 5665 |
| 24 | TI "health worker*" OR AB "health worker*" | 11661 |

***Table Three:*** Continued.

| 25 | TI "professionals" OR AB "professionals" | 133741 |
| --- | --- | --- |
| 26 | TI "healthcare" OR AB "healthcare" | 196033 |
| 27 | TI "care" OR AB "care" | 1014125 |
| 28 | TI "therapy" OR AB "therapy" | 489284 |
| 29 | TI "treatment" OR AB "treatment" | 1008569 |
| 30 | 14 or 15 or 16 or 17 or 18 or 19 or 20 or 21 or 22 or 23 or 24 or 25 or 26 or 27 or 28 or 29  (MH "Health Care Delivery+") OR (MH "Right to Health") OR (MH "Patient Advocacy") OR (MH "Physician-Patient Relations") OR (MH "Health Personnel+") OR TI "personnel" OR AB "personnel" OR TI "physician*" OR AB "physician*" OR TI "doctor*" OR AB "doctor*" OR TI "nurse*" OR AB "nurse*" OR TI "health provider*" OR AB "health provider*" OR TI "health worker*" OR AB "health worker*" OR TI "professionals" OR AB "professionals" OR TI "healthcare" OR AB "healthcare" OR TI "care" OR AB "care" OR TI "therapy" OR AB "therapy" OR TI "treatment" OR AB "treatment" | 3020536 |
| ***Third search block: Outcome (ethical challenges)*** | | |
| 31 | (MH "Ethics+") | 133898 |
| 32 | (MH "Ethics, Medical") | 11958 |
| 33 | (MH "Ethics, Nursing") | 10819 |
| 34 | (MH "Ethics Committees") | 2274 |
| 35 | (MH "Ethics, Organizational") | 688 |
| 36 | (MH "Jurisprudence+") | 94415 |
| 37 | (MH "Ethics Theory+") | 863 |
| 38 | TI "ethic*" OR AB "ethic*" | 75546 |
| 39 | TI "bioethic*" OR AB "bioethic*" | 5180 |
| 40 | TI "moral*" OR AB "moral*" | 21194 |
| 41 | TI "professional obligation*" OR AB "professional obligation*" | 378 |
| 42 | TI "responsabilit*" OR AB "responsabilit*" | 339 |
| 43 | TI "issue*" OR AB "issue*" | 333688 |
| 44 | TI "challenge*" OR AB "challenge*" | 240271 |
| 45 | TI "dilemma*" OR AB "dilemma*" | 17605 |

***Table Three:*** Continued.

| 46 | 31 or 32 or 33 or 34 or 35 or 36 or 37 or 38 or 39 or 40 or 41 or 42 or 43 or 44 or 45  (MH "Ethics+") OR (MH "Ethics, Medical") OR (MH "Ethics, Nursing") OR (MH "Ethics Committees") OR (MH "Ethics, Organizational") OR (MH "Jurisprudence+") OR (MH "Ethics Theory+") OR TI "ethic*" OR AB "ethic*" OR TI "bioethic*" OR AB "bioethic*" OR TI "moral*" OR AB "moral*" OR TI "professional obligation*" OR AB "professional obligation*" OR TI "responsabilit*" OR AB "responsabilit*" OR TI "issue*" OR AB "issue*" OR TI "challenge*" OR AB "challenge*" OR TI "dilemma*" OR AB "dilemma*" | 777145 |
| --- | --- | --- |
| ***Final search query: Combining the three search blocks by Boolean operator “and”*** | | |
| 47 | 13 and 30 and 46  ((MH "Undocumented Immigrants") OR TI "undocumented" OR AB "undocumented" OR TI "unauthorized immigrant*" OR AB "unauthorized immigrant*" OR TI "unauthorized migrant*" OR AB "unauthorized migrant*" OR TI "unauthorized worker*" OR AB "unauthorized worker*" OR TI "illegal immigrant*" OR AB "illegal immigrant*" OR TI "illegal migrant*" OR AB "illegal migrant*" OR TI "illegal worker*" OR AB "illegal worker*" OR TI "irregular migrant*" OR AB "irregular migrant*" OR TI "irregular immigrant*" OR AB "irregular immigrant*" OR TI "irregular worker*" OR AB "irregular worker*" OR TI "rejected asylum seeker*" OR AB "rejected asylum seeker*") AND ((MH "Health Care Delivery+") OR (MH "Right to Health") OR (MH "Patient Advocacy") OR (MH "Physician-Patient Relations") OR (MH "Health Personnel+") OR TI "personnel" OR AB "personnel" OR TI "physician*" OR AB "physician*" OR TI "doctor*" OR AB "doctor*" OR TI "nurse*" OR AB "nurse*" OR TI "health provider*" OR AB "health provider*" OR TI "health worker*" OR AB "health worker*" OR TI "professionals" OR AB "professionals" OR TI "healthcare" OR AB "healthcare" OR TI "care" OR AB "care" OR TI "therapy" OR AB "therapy" OR TI "treatment" OR AB "treatment") AND ((MH "Ethics+") OR (MH "Ethics, Medical") OR (MH "Ethics, Nursing") OR (MH "Ethics Committees") OR (MH "Ethics, Organizational") OR (MH "Jurisprudence+") OR (MH "Ethics Theory+") OR TI "ethic*" OR AB "ethic*" OR TI "bioethic*" OR AB "bioethic*" OR TI "moral*" OR AB "moral*" OR TI "professional obligation*" OR AB "professional obligation*" OR TI "responsabilit*" OR AB "responsabilit*" OR TI "issue*" OR AB "issue*" OR TI "challenge*" OR AB "challenge*" OR TI "dilemma*" OR AB "dilemma*") | 472 |
| 48 | limit 47 to English language | 466 |
| Note: MH = Cinahl subject heading, + = exploded subject heading, TI = title and AB = abstract  The asterisk (*) is used for truncation  Double quotation marks (“ ”) around a phrase refer that the words should be present in the same sequence as in the search phrase  The Boolean operator (or) is used to expand a search by broadening the set and indicates that each result contains at least one search term  The Boolean operator (and) is used to narrow a search by linking different concepts or search blocks and indicates that each result contains all search terms or queries | | |

***Table Four:*** Search strategy by using Cochrane Library and the total number of retrieved records for each search query categorized according to the main search blocks (population, intervention, and outcome). Date of search: 12-Mar-23

| **No.** | **Search query** | **Total no. of retrieved records** |
| --- | --- | --- |
| ***First search block: Population (undocumented migrants)*** | | |
| 1 | MeSH descriptor: [Undocumented Immigrants] explode all trees | 2 |
| 2 | (undocumented):ti,ab,kw | 90 |
| 3 | (unauthorized immigrant*):ti,ab,kw | 1 |
| 4 | (unauthorized migrant*):ti,ab,kw | 0 |
| 5 | (unauthorized worker*):ti,ab,kw | 9 |
| 6 | (illegal immigrant*):ti,ab,kw | 3 |
| 7 | (illegal migrant*):ti,ab,kw | 4 |
| 8 | (illegal worker*):ti,ab,kw | 15 |
| 9 | (irregular migrant*):ti,ab,kw | 0 |
| 10 | (irregular immigrant*):ti,ab,kw | 1 |
| 11 | (irregular worker*):ti,ab,kw | 50 |
| 12 | (rejected asylum seeker*):ti,ab,kw | 0 |
| 13 | 1 or 2 or 3 or 4 or 5 or 6 or 7 or 8 or 9 or 10 or 11 or 12 | 168 |
| ***Second search block: Intervention (Patient advocacy strategies employed by healthcare providers in the process of delivering healthcare services)*** | | |
| 14 | MeSH descriptor: [Delivery of Health Care] explode all trees | 57532 |
| 15 | MeSH descriptor: [Right to Health] explode all trees | 0 |
| 16 | MeSH descriptor: [Patient Advocacy] explode all trees | 127 |
| 17 | MeSH descriptor: [Physician-Patient Relations] explode all trees | 1734 |
| 18 | MeSH descriptor: [Health Personnel] explode all trees | 13032 |
| 19 | (personnel):ti,ab,kw | 18169 |
| 20 | (physician*):ti,ab,kw | 52362 |
| 21 | (doctor*):ti,ab,kw | 17942 |
| 22 | (nurse*):ti,ab,kw | 31802 |
| 23 | (health provider*):ti,ab,kw | 14535 |
| 24 | (health worker*):ti,ab,kw | 9593 |
| 25 | (professionals):ti,ab,kw | 27203 |
| 26 | (healthcare):ti,ab,kw | 27602 |
| 27 | (care):ti,ab,kw | 286047 |
| 28 | (therapy):ti,ab,kw | 808095 |
| 29 | (treatment):ti,ab,kw | 903748 |
| 30 | 14 or 15 or 16 or 17 or 18 or 19 or 20 or 21 or 22 or 23 or 24 or 25 or 26 or 27 or 28 or 29 | 1295273 |

***Table Four:*** Continued.

| ***Third search block: Outcome (ethical challenges)*** | | |
| --- | --- | --- |
| 31 | MeSH descriptor: [Ethics] explode all trees | 1393 |
| 32 | MeSH descriptor: [Morals] explode all trees | 35 |
| 33 | MeSH descriptor: [Jurisprudence] explode all trees | 2644 |
| 34 | (ethic*):ti,ab,kw | 23107 |
| 35 | (bioethic*):ti,ab,kw | 231 |
| 36 | (moral*):ti,ab,kw | 1116 |
| 37 | (professional obligation*):ti,ab,kw | 59 |
| 38 | (responsabilit*):ti,ab,kw | 32 |
| 39 | (issue*):ti,ab,kw | 32395 |
| 40 | (challenge*):ti,ab,kw | 34414 |
| 41 | (dilemma*):ti,ab,kw | 886 |
| 42 | 31 or 32 or 33 or 34 or 35 or 36 or 37 or 38 or 39 or 40 or 41 | 89086 |
| ***Final search query: Combining the three search blocks by Boolean operator “and”*** | | |
| 43 | 13 and 30 and 42 | 27 |
| MeSH = Medical subject headings, ti = title, ab = abstract, and kw = keyword heading  The asterisk (*) is used for truncation  The Boolean operator (or) is used to expand a search by broadening the set and indicates that each result contains at least one search term  The Boolean operator (and) is used to narrow a search by linking different concepts or search blocks and indicates that each result contains all search terms or queries | | |

***Table Five:*** The additional number of retrieved records by employing the same search query across the four selected databases and updating the search date to May 20, 2024.

| **Database** | **Total number of retrieved records according to the search date** | | **Additional number of retrieved records** |
| --- | --- | --- | --- |
|  | 12-Mar-23 | 20-May-24 |  |
| Medline/Ovid | 571 | 629 | 58 |
| Embase | 868 | 1035 | 167 |
| Cinahl | 466 | 490 | 24 |
| Cochrane Library | 27 | 28 | 1 |
| Cumulative total of retrieved records | **1932** | **2182** | **250** |
| Cumulative total after excluding duplicate records | **1201** | **1343** | **142** |
