# Supplementary material for: Healthcare providers' advocacy approaches and ethical challenges in delivering healthcare to undocumented migrants: a scoping review
Source: Med Health Care Philos. 2024 Oct 7;27(4):579–606. doi: 10.1007/s11019-024-10225-8 (PMC11519158; doi:10.1007/s11019-024-10225-8)
Supplement: Supplementary file 2 — Supplementary file2 (DOCX 18 KB) [file 11019_2024_10225_MOESM2_ESM.docx]

***Appendix Two:*** Search strings used for each search block (concept) based on our PICO framework and the final search queries stratified by the databases*

| ***Database*** | ***First search block: Undocumented migrants*** |  | ***Second search block: Patient advocacy strategies during the healthcare delivery process*** |  | ***Third search block: Ethical challenges*** |
| --- | --- | --- | --- | --- | --- |
| ***Medline*** | exp undocumented immigrants/ or undocumented.ab,kw,ti. or unauthorized immigrant$.ab,kw,ti. or unauthorized migrant$.ab,kw,ti. or unauthorized worker$.ab,kw,ti. or illegal immigrant$.ab,kw,ti. or illegal migrant$.ab,kw,ti. or illegal worker$.ab,kw,ti. or irregular migrant$.ab,kw,ti. or irregular immigrant$.ab,kw,ti. or irregular worker$.ab,kw,ti. or rejected asylum seeker$.ab,kw,ti. | **AND** | exp "delivery of health care"/ or exp health services accessibility/ or exp healthcare disparities/ or exp right to health/ or exp patient advocacy/ or exp physician-patient relations/ or exp health personnel/ or exp physicians/ or exp allied health personnel/ or personnel.ab,kw,ti. or physician$.ab,kw,ti. or doctor$.ab,kw,ti. or nurse$.ab,kw,ti. or health provider$.ab,kw,ti. or health worker$.ab,kw,ti. or professionals.ab,kw,ti. or healthcare.ab,kw,ti. or care.ab,kw,ti. or therapy.ab,kw,ti. or treatment.ab,kw,ti. | **AND** | exp ethics/ or exp bioethics/ or exp moral obligations/ or exp ethics, clinical/ or exp ethics, institutional/ or exp jurisprudence/ or exp ethics committees, clinical/ or exp "codes of ethics"/ or exp ethics consultation/ or exp principle-based ethics/ or exp ethics committees/ or exp ethics, professional/ or exp ethics, nursing/ or exp ethics, medical/ or exp ethical theory/ or ethic$.ab,kw,ti. or bioethic$.ab,kw,ti. or moral$.ab,kw,ti. or "professional obligation$".ab,kw,ti. or responsabilit$.ab,kw,ti. or issue$.ab,kw,ti. or challenge$.ab,kw,ti. or dilemma$.ab,kw,ti. |
| ***Final search query*** | (exp undocumented immigrants/ or undocumented.ab,kw,ti. or unauthorized immigrant$.ab,kw,ti. or unauthorized migrant$.ab,kw,ti. or unauthorized worker$.ab,kw,ti. or illegal immigrant$.ab,kw,ti. or illegal migrant$.ab,kw,ti. or illegal worker$.ab,kw,ti. or irregular migrant$.ab,kw,ti. or irregular immigrant$.ab,kw,ti. or irregular worker$.ab,kw,ti. or rejected asylum seeker$.ab,kw,ti.) and (exp "delivery of health care"/ or exp health services accessibility/ or exp healthcare disparities/ or exp right to health/ or exp patient advocacy/ or exp physician-patient relations/ or exp health personnel/ or exp physicians/ or exp allied health personnel/ or personnel.ab,kw,ti. or physician$.ab,kw,ti. or doctor$.ab,kw,ti. or nurse$.ab,kw,ti. or health provider$.ab,kw,ti. or health worker$.ab,kw,ti. or professionals.ab,kw,ti. or healthcare.ab,kw,ti. or care.ab,kw,ti. or therapy.ab,kw,ti. or treatment.ab,kw,ti.) and (exp ethics/ or exp bioethics/ or exp moral obligations/ or exp ethics, clinical/ or exp ethics, institutional/ or exp jurisprudence/ or exp ethics committees, clinical/ or exp "codes of ethics"/ or exp ethics consultation/ or exp principle-based ethics/ or exp ethics committees/ or exp ethics, professional/ or exp ethics, nursing/ or exp ethics, medical/ or exp ethical theory/ or ethic$.ab,kw,ti. or bioethic$.ab,kw,ti. or moral$.ab,kw,ti. or "professional obligation$".ab,kw,ti. or responsabilit$.ab,kw,ti. or issue$.ab,kw,ti. or challenge$.ab,kw,ti. or dilemma$.ab,kw,ti.) | | | | |
| ***Embase*** | 'undocumented immigrant'/exp OR undocumented:ab,ti OR 'unauthorized immigrant*':ab,ti OR 'unauthorized migrant*':ab,ti OR 'unauthorized worker*':ab,ti OR 'illegal immigrant*':ab,ti OR 'illegal migrant*':ab,ti OR 'illegal worker*':ab,ti OR 'irregular migrant*':ab,ti OR 'irregular immigrant*':ab,ti OR 'irregular worker*':ab,ti OR 'rejected asylum seeker*':ab,ti | **AND** | 'health care delivery'/exp OR 'health care disparity'/exp OR 'patient advocacy'/exp OR 'professional-patient relationship'/exp OR 'health care personnel'/exp OR personnel:ab,ti OR 'physician*':ab,ti OR 'doctor*':ab,ti OR 'nurse*':ab,ti OR 'health provider*':ab,ti OR 'health worker*':ab,ti OR professionals:ab,ti OR healthcare:ab,ti OR care:ab,ti OR therapy:ab,ti OR treatment:ab,ti | **AND** | 'ethics'/exp OR 'professional standard'/exp OR 'morality'/exp OR 'jurisprudence'/exp OR ethic*:ab,ti OR bioethic*:ab,ti OR moral*:ab,ti OR 'professional obligation*':ab,ti OR responsabilit*:ab,ti OR issue*:ab,ti OR challenge*:ab,ti OR dilemma*:ab,ti |
| ***Final search query*** | ('undocumented immigrant'/exp OR undocumented:ab,ti OR 'unauthorized immigrant*':ab,ti OR 'unauthorized migrant*':ab,ti OR 'unauthorized worker*':ab,ti OR 'illegal immigrant*':ab,ti OR 'illegal migrant*':ab,ti OR 'illegal worker*':ab,ti OR 'irregular migrant*':ab,ti OR 'irregular immigrant*':ab,ti OR 'irregular worker*':ab,ti OR 'rejected asylum seeker*':ab,ti) AND ('health care delivery'/exp OR 'health care disparity'/exp OR 'patient advocacy'/exp OR 'professional-patient relationship'/exp OR 'health care personnel'/exp OR personnel:ab,ti OR 'physician*':ab,ti OR 'doctor*':ab,ti OR 'nurse*':ab,ti OR 'health provider*':ab,ti OR 'health worker*':ab,ti OR professionals:ab,ti OR healthcare:ab,ti OR care:ab,ti OR therapy:ab,ti OR treatment:ab,ti) AND ('ethics'/exp OR 'professional standard'/exp OR 'morality'/exp OR 'jurisprudence'/exp OR ethic*:ab,ti OR bioethic*:ab,ti OR moral*:ab,ti OR 'professional obligation*':ab,ti OR responsabilit*:ab,ti OR issue*:ab,ti OR challenge*:ab,ti OR dilemma*:ab,ti) | | | | |
| ***Cinahl*** | (MH "Undocumented Immigrants") OR TI "undocumented" OR AB "undocumented" OR TI "unauthorized immigrant*" OR AB "unauthorized immigrant*" OR TI "unauthorized migrant*" OR AB "unauthorized migrant*" OR TI "unauthorized worker*" OR AB "unauthorized worker*" OR TI "illegal immigrant*" OR AB "illegal immigrant*" OR TI "illegal migrant*" OR AB "illegal migrant*" OR TI "illegal worker*" OR AB "illegal worker*" OR TI "irregular migrant*" OR AB "irregular migrant*" OR TI "irregular immigrant*" OR AB "irregular immigrant*" OR TI "irregular worker*" OR AB "irregular worker*" OR TI "rejected asylum seeker*" OR AB "rejected asylum seeker*" | **AND** | (MH "Health Care Delivery+") OR (MH "Right to Health") OR (MH "Patient Advocacy") OR (MH "Physician-Patient Relations") OR (MH "Health Personnel+") OR TI "personnel" OR AB "personnel" OR TI "physician*" OR AB "physician*" OR TI "doctor*" OR AB "doctor*" OR TI "nurse*" OR AB "nurse*" OR TI "health provider*" OR AB "health provider*" OR TI "health worker*" OR AB "health worker*" OR TI "professionals" OR AB "professionals" OR TI "healthcare" OR AB "healthcare" OR TI "care" OR AB "care" OR TI "therapy" OR AB "therapy" OR TI "treatment" OR AB "treatment" | **AND** | (MH "Ethics+") OR (MH "Ethics, Medical") OR (MH "Ethics, Nursing") OR (MH "Ethics Committees") OR (MH "Ethics, Organizational") OR (MH "Jurisprudence+") OR (MH "Ethics Theory+") OR TI "ethic*" OR AB "ethic*" OR TI "bioethic*" OR AB "bioethic*" OR TI "moral*" OR AB "moral*" OR TI "professional obligation*" OR AB "professional obligation*" OR TI "responsabilit*" OR AB "responsabilit*" OR TI "issue*" OR AB "issue*" OR TI "challenge*" OR AB "challenge*" OR TI "dilemma*" OR AB "dilemma*" |
| ***Final search query*** | ((MH "Undocumented Immigrants") OR TI "undocumented" OR AB "undocumented" OR TI "unauthorized immigrant*" OR AB "unauthorized immigrant*" OR TI "unauthorized migrant*" OR AB "unauthorized migrant*" OR TI "unauthorized worker*" OR AB "unauthorized worker*" OR TI "illegal immigrant*" OR AB "illegal immigrant*" OR TI "illegal migrant*" OR AB "illegal migrant*" OR TI "illegal worker*" OR AB "illegal worker*" OR TI "irregular migrant*" OR AB "irregular migrant*" OR TI "irregular immigrant*" OR AB "irregular immigrant*" OR TI "irregular worker*" OR AB "irregular worker*" OR TI "rejected asylum seeker*" OR AB "rejected asylum seeker*") AND ((MH "Health Care Delivery+") OR (MH "Right to Health") OR (MH "Patient Advocacy") OR (MH "Physician-Patient Relations") OR (MH "Health Personnel+") OR TI "personnel" OR AB "personnel" OR TI "physician*" OR AB "physician*" OR TI "doctor*" OR AB "doctor*" OR TI "nurse*" OR AB "nurse*" OR TI "health provider*" OR AB "health provider*" OR TI "health worker*" OR AB "health worker*" OR TI "professionals" OR AB "professionals" OR TI "healthcare" OR AB "healthcare" OR TI "care" OR AB "care" OR TI "therapy" OR AB "therapy" OR TI "treatment" OR AB "treatment") AND ((MH "Ethics+") OR (MH "Ethics, Medical") OR (MH "Ethics, Nursing") OR (MH "Ethics Committees") OR (MH "Ethics, Organizational") OR (MH "Jurisprudence+") OR (MH "Ethics Theory+") OR TI "ethic*" OR AB "ethic*" OR TI "bioethic*" OR AB "bioethic*" OR TI "moral*" OR AB "moral*" OR TI "professional obligation*" OR AB "professional obligation*" OR TI "responsabilit*" OR AB "responsabilit*" OR TI "issue*" OR AB "issue*" OR TI "challenge*" OR AB "challenge*" OR TI "dilemma*" OR AB "dilemma*") | | | | |
| *Review appendix one for the detailed search strategy including the search terms that were used in the Cochrane Library | | | | | |
