# Supplementary material for: Healthcare providers' advocacy approaches and ethical challenges in delivering healthcare to undocumented migrants: a scoping review
Source: Med Health Care Philos. 2024 Oct 7;27(4):579–606. doi: 10.1007/s11019-024-10225-8 (PMC11519158; doi:10.1007/s11019-024-10225-8)
Supplement: Supplementary file 3 — Supplementary file3 (DOCX 17 KB) [file 11019_2024_10225_MOESM3_ESM.docx]

**Data Extraction Form**

| 1 | ***Year of publication:*** |
| --- | --- |
| 2 | ***Reference:*** |
| 3 | ***First author:*** |
| 4 | ***Study location:*** |
| 5 | ***Aim of study:*** |
| 6 | ***Design:***  □ Qualitative  □ Mixed |
| 7 | ***Methods:***  □ Interviews (semi-structured)  □ Interviews (in-depth)  □ Interviews (both in-depth and semi-structured)  □ Ethnographic fieldwork (direct observation & interviews)  □ Focus group discussions  □ Semi-structured interviews and focus group discussions  □ In-depth interviews and focus group discussions |
| 8 | ***Health sector or service delivery:***  □ Public sector (formal or governmental)  □ Private sector (non-governmental or humanitarian)  □ Both (governmental and humanitarian)  □ Unspecified |
| 9 | ***Health field or discipline:***  □ Emergency medicine  □ Maternal health  □ Child health  □ Mental health  □ Primary care  □ Oral health  □ Oncology  □ Interdisciplinary  □ Unspecified  □ General practice  □ Organ transplantation |
| 10 | ***Sample size:*** |
| 11 | ***Healthcare professions only:***  □ Yes  □ No |
| 12 | ***Physicians only:***  □ Yes  □ No |
| 13 | ***Nurses only:***  □ Yes  □ No |
| 14 | ***Classification of healthcare providers according to profession:***  □ Unspecified  □ Physicians  □ Clinicians  □ Nurses  □ Physician assistants  □ Nurse practitioners  □ Nurse assistants  □ Healthcare, medical evaluation, or clinical assistants  □ Dietitians  □ Community health workers  □ Social workers  □ Early childhood specialists  □ Cultural mediators  □ Medical students  □ Senior executives  □ First aid workers  □ Administrators  □ Service representatives/specialists  □ Mental health professionals and experts  □ Dentists  □ NGO and CSO staff |
| Note: The values for variables No. 7, 9, and 14 were consistently added as required | |
