# Supplementary material for: Healthcare providers' advocacy approaches and ethical challenges in delivering healthcare to undocumented migrants: a scoping review
Source: Med Health Care Philos. 2024 Oct 7;27(4):579–606. doi: 10.1007/s11019-024-10225-8 (PMC11519158; doi:10.1007/s11019-024-10225-8)
Supplement: Supplementary file 4 — Supplementary file4 (DOCX 37 KB) [file 11019_2024_10225_MOESM4_ESM.docx]

***Appendix Three*:** Summary of the qualitative papers included (n = 30), wherein healthcare providers (n = 915) have engaged in various advocacy activities to bolster the provision of healthcare services for undocumented migrants.

| **No.** | **Study & Location** | **Aim** | **Design &**  **Methods** | **Health**  **Sector or Service Delivery** | **Health Field or Discipline** | **Sample Size** | **Classification of Participants According to Profession** |
| --- | --- | --- | --- | --- | --- | --- | --- |
| 1 | Biswas et al. (2011)*  Denmark | To describe and analyze UMs’ experiences of access to healthcare, use of alternative health-seeking strategies, and ER nurses’ experiences in encounters with UMs | Qualitative: Ethnographic fieldwork, direct observation & interviews | Public sector (formal or governmental) | EM | 8 | - 8 nurses |
| 2 | Castaneda. (2011)*^┼^  Germany | To present the viewpoints of physicians engaged in medical humanitarianism and discuss the complications associated with filling access gaps through NGOs | Qualitative: Ethnographic fieldwork, direct observation & interviews | Private sector (non-governmental or humanitarian) | Unspecified | 61 | - 61 interviews were conducted with migrants and volunteer staff from NGOs, including physicians |
| 3 | Jensen et al. (2011)  Denmark | To explore how health professionals navigate and how they experience providing treatment for UMs in the Danish healthcare system | Qualitative: Semi-structured, vignette-based interviews | Public sector (formal or governmental) | Interdisciplinary:   - General practice - EM | 12 | - 12 physicians |
| 4 | Willen. (2011)*  Israel | To make sense of how “illegal” migration status is configured by the Israeli state and civil society and, moreover, how this rapidly evolving form of legal (non)classification shapes and constrains im/migrants’ embodied experiences of health, illness., pregnancy, and reproduction, as well as their broader experiences of subjectivity, morality, and being-in-the-world | Qualitative: Ethnographic fieldwork, direct observation & interviews | Private sector (non-governmental or humanitarian) | Interdisciplinary:   - Maternal health - General practice - Specialty care | 25 | - 25 staff members and volunteers |
| 5 | Dauvrin et al. (2012)  Multiple locations | To explore the experiences of health professionals providing care to irregular migrants in three types of healthcare service (primary care, mental health, and accident and emergency care) across 16 European countries | Qualitative: Semi-structured, vignette-based interviews | Unspecified | Interdisciplinary:   - Primary care - Mental health - EM | 240 | - 240 unspecified |
| 6 | Holmes. (2012)*  United States | To explore the sociocultural factors affecting the interactions and barriers between U.S. biomedical professionals and their unauthorized Mexican migrant patients | Qualitative: Ethnographic fieldwork, direct observation & interviews | Public sector (formal or governmental) | Unspecified | 30 | - 18 physicians - 10 nurses - 2 dentists |
| 7 | Marrow. (2012)  United States | To examine how one uniquely inclusive American local policy climate affects the attitudes and behaviors of public safety-net healthcare providers towards UMs, and thus potentially by extension, UMs’ access to and utilization of healthcare | Qualitative: Semi-structured interviews | Public sector (formal or governmental) | Primary care | 36 | - 8 registered nurses - 7 resident physicians-in-training - 7 medical evaluation assistants - 5 physicians - 4 clerical staff - 3 nurse practitioners - 1 social worker - 1 health worker |
| 8 | Straβmayr et al. (2012)  Multiple locations | To identify barriers to mental healthcare for irregular migrants, and to explore ways by which these barriers are overcome in practice | Qualitative: Face-to-face, semi-structured, vignette-based interviews | Both (governmental and humanitarian) | Mental health | 25 | - 25 mental health experts |
| 9 | Gullberg et al. (2014)  Sweden | To investigate how nurses working in emergency healthcare in Sweden experience their encounter with UMs | Qualitative: Semi-structured interviews | Both (governmental and humanitarian) | Interdisciplinary:   - EM (major, psychiatric, and women’s emergency) - Primary care | 16 | - 16 nurses |
| 10 | Tiedje et al. (2014)  United States | To discuss immigration, health, and human rights while examining solidarity, spirituality, and advocacy using a U.S.-based example of medical humanitarianism; the “Our Lady of Guadalupe Free Clinic” | Qualitative: Ethnographic fieldwork, direct observation & interviews | Private sector (non-governmental or humanitarian) | Primary care | 30 | - 30 clinic volunteers (health professionals, administrators, language interpreters, and spiritual leaders) |
| 11 | Teunissen et al. (2015)  Netherlands | To explore the views and experiences of general practitioners in relation to recognition, recording, and treatment of mental health problems of UMs | Qualitative: Semi-structured interviews | Public sector (formal or governmental) | Mental health | 16 | - 16 physicians |
| 12 | Sandblom et al. (2017)  Sweden | To illuminate the experience of nurses providing healthcare to UMs in a voluntary network | Qualitative: Semi-structured interviews | Private sector (non-governmental or humanitarian) | Unspecified | 7 | - 7 nurses |
| 13 | Cervantes et al. (2018)  United States | To understand clinicians’ experiences providing emergency-only hemodialysis for UMs with end-stage kidney disease | Qualitative: One-to-one, in-person, semi-structured interviews | Public sector (formal or governmental) | Interdisciplinary:   - EM - IM - Nephrology - Palliative care - Social work - Nutrition | 50 | - 27 physicians - 16 nurses - 3 physician assistants - 2 nurse assistants - 1 dietitian - 1 social worker |
| 14 | Armin. (2019)  United States | To describe the work of a variety of healthcare staff who manage specialized cancer for publicly insured patients who have difficulty gaining or maintaining program eligibility or for uninsured and undocumented patients who are excluded from state programs | Qualitative: Ethnographic fieldwork, direct observation & interviews | Both (governmental and private) | Oncology (breast cancer care) | 33 | - 13 administrators - 6 physicians - 6 nurses - 5 patient advocates/community health workers - 3 social workers/behavioral health professionals |
| 15 | Bianchi et al. (2019)  United States | To explore attitudes about UMs’ deservingness of healthcare among staff and professional health workers | Qualitative: in-depth, semi-structured  interviews | Public sector (formal or governmental) | Unspecified | 31 | - 11 clinical assistants - 9 social service coordinators - 7 clerical staff - 2 Institutional resource managers - 2 Clinicians |
| 16 | Fabi et al. (2019)  United States | To characterize the professional practice norms embraced by providers within various prenatal policy environments, and to explore the ethical tensions they encounter while striving to uphold these norms | Qualitative: in-depth, semi-structured phone interviews | Public sector (formal or governmental) | Maternal health | 34 | - 12 social workers, or patient support workers (e.g., prenatal educator, or outreach team member) - 8 primary obstetrical providers (e.g., obstetrician, family doctor, or midwife) - 7 nurses, or other providers (e.g., mental health clinician) - 7 billing or clinic administrators |
| 17 | Lopez-Domene et al. (2019)*  Spain | To describe and understand the experiences and health needs of women irregular migrants during emergency care provision upon arrival in Spain by small boat | Qualitative: In-depth interviews | Private sector (non-governmental or humanitarian) | EM (women’s emergency with special focus on sexual exploitation and human trafficking) | 10 | - 4 nurses - 3 cultural mediators - 2 maritime rescue captains - 1 psychologist |
| 18 | Doshi et al. (2020)  United States | To examine barriers and facilitators to healthcare and social services among undocumented Latino(a)/Latinx immigrants in Southeast Michigan from the perspective of frontline service providers | Qualitative: in-depth, semi-structured  interviews | Both (governmental and humanitarian) | Unspecified | 28 | - 10 service representatives/specialists - 6 community health workers/advocates - 5 clinical practitioners - 5 administrators - 2 other |
| 19 | Granero-Molina et al. (2021)  Spain | To describe and understand the experiences of physicians in emergency care for UMs who arrive in Spain by small boats | Qualitative: In-depth interviews | Private sector (non-governmental or humanitarian) | Interdisciplinary:   - FM - Pediatrics - Geriatrics - Tropical medicine - Forensic Medicine - Oral medicine | 16 | - 16 physicians |
| 20 | Hoekstra. (2021)  United States | To argue for the recognition of medical provision for immigrants as healthcare advocacy, while emphasizing the role of local and national immigration and health policies in shaping the relationship between healthcare provision and political advocacy, through an examination of the medical humanitarian initiatives of a community clinic in Arizona | Qualitative: Ethnographic fieldwork, direct observation & interviews | Private sector (non-governmental or humanitarian) | Interdisciplinary:   - Primary care - Women’s & maternal health - Mental health - Allied health (physical therapy) - Naturopathy | 35 | - 35 key informants (founders, board members, staff, and volunteers) who are engaged in diverse roles at the community clinic |
| 21 | Lafaut. (2021)  Belgium | To understand how individual healthcare workers who regularly take care of undocumented migrants deal with ethical dilemmas in practice | Qualitative: Ethnographic fieldwork, direct observation & interviews | Both (governmental and humanitarian) | Unspecified | 45 | - 45 unspecified |
| 22 | Midde et al. (2021)*  Netherlands | To explore the accessibility of a voluntary dental network providing dental treatments to UMs in the Netherlands from the perspectives of patients, dentists, and staff members of NGOs | Qualitative: Semi-structured interviews | Private sector (non-governmental or humanitarian) | Oral health | 9 | - 7 dentists - 2 staff members of NGOs |
| 23 | Saadi et al. (2021)  United States | To identify factors enabling or challenging the implementation of interventions aimed at mitigating immigration-related stressors in the healthcare context | Qualitative: Semi-structured interviews | Both (governmental and private) | Unspecified | 38 | - 38 stakeholders involved in implementation of interventions including providers in clinical and/or administrative roles and senior executives |
| 24 | Granero-Molina et al. (2022)  Spain | To describe and understand the experiences of nurses providing emergency care to UMs who arrive in Spain in small boats | Qualitative: Face-to-face, in-depth interviews | Private sector (non-governmental or humanitarian) | EM | 17 | - 17 nurses |
| 25 | Kvamme et al. (2022)  Norway | To describe how public health nurses experienced challenges and dilemmas in ensuring the best interests of the undocumented migrant child | Qualitative: Semi-structured & focus group interviews | Public sector (formal or governmental) | Child health | 7 | - 7 nurses |
| 26 | Vanobberghen et al. (2022)  Belgium | To describe the experiences, motivations, and concerns of the health professionals during the medical monitoring of the hunger strike in a non-custodial setting | Qualitative: Focus group discussions | Private sector (non-governmental or humanitarian) | Interdisciplinary:   - IM - FM | 18 | - 11 physicians - 2 nurses - 5 medical students |
| 27 | Mladovsky. (2023)  England | To explore the everyday governance of the mental health coverage for forced migrants in the English NHS and NGO sector | Qualitative: Ethnographic fieldwork, direct observation & interviews | Both (governmental and humanitarian) | Mental health | 25 | - 25 mental health professionals (clinical psychologists, psychiatrists, psychotherapists, psychoanalysts, counsellors, mental health commissioners, and policymakers) |
| 28 | Jiménez-Lasserrotte et al. (2023)  Spain | To describe and understand the experiences of healthcare providers in relation to the healthcare needs and the process of emergency care for child irregular migrants who come to Spain in small vessels | Qualitative: In-depth interviews & focus group discussions | Private sector (non-governmental or humanitarian) | Interdisciplinary:   - EM - Child health | 21 | - 8 nurses - 5 cultural mediators - 3 social workers - 3 early childhood specialists   2 team leaders |
| 29 | Gely et al. (2023)*  United States | To understand how access to kidney transplantation affects patients, their family, healthcare providers, and the healthcare system | Qualitative: Semi-structured interviews | Public sector (formal or governmental) | Organ transplantation (kidney transplantation) | 13 | - 5 physicians (nephrologists and transplant surgeons) - 4 transplant center professionals (nurses, financial counselors and social workers) - 4 community outreach stakeholders |
| 30 | Piccoli et al. (2024)  Multiple locations | To explore the ethical dilemmas faced by CSOs when providing healthcare services to irregular migrants in countries with universal healthcare provisions, and to investigate the conditions under which these ethical dilemmas occur, as well as the strategies CSO staff use to mitigate them | Qualitative: in-depth, semi-structured  interviews | Private sector (non-governmental or humanitarian) | Interdisciplinary | 40 | - 40 CSO staff (doctors, nurses, and other professionals, including social workers, support staff, and cultural mediators) |
| Note: UMs = Undocumented Migrants, ER = Emergency Room, EM = Emergency Medicine, IM = Internal Medicine, FM = Family Medicine, NHS = National Health Service, NGO = Non-governmental organization, CSO = Civil Society Organization  Note: The figure of 915 healthcare providers signifies the combined count of healthcare professionals interviewed in the qualitative studies encompassed in our scoping review. However, it's important to note that this total doesn't necessarily reflect the overall number of healthcare providers engaged in advocacy activities for undocumented migrants during healthcare delivery, as determining this specific number poses a challenge.  *The study samples in these studies encompassed both healthcare providers and undocumented migrants. However, our analysis specifically concentrated on the data derived from healthcare providers.  ^┼^The sample size of 61 participants in the study refers to the total number of interviewed undocumented migrants and healthcare providers combined. Consequently, it was not considered in the overall count of healthcare providers included in our paper, given that the specific number of healthcare providers involved in this study is not explicitly stated. | | | | | | | |
